# PI-Mamba: linear-time protein backbone generation via spectrally initialized flow matching

**DOI:** 10.1093/bioinformatics/btag370

**Published:** 2026-08-21

**Authors:** Tianyu Wu, Lin Zhu

**Affiliations:** Center for Biophysics and Quantitative Biology, University of Illinois Urbana-Champaign, Urbana, IL 61801, United States; School of Information Science, University of Illinois Urbana-Champaign, Champaign, IL 61820, United States

## Abstract

**Motivation:**

Generative models for protein backbone design have to simultaneously ensure geometric validity, sampling efficiency, and scalability to long protein chains. However, most existing approaches rely on iterative refinement, quadratic attention mechanisms, or post-hoc geometry correction, leading to a persistent trade-off between computational efficiency and structural fidelity.

**Results:**

We present Physics-Informed Mamba (PI-Mamba), a generative model that enforces exact local covalent geometry by construction while enabling linear-time inference. PI-Mamba integrates a differentiable constraint-enforcement operator into a flow-matching framework and couples it with a Mamba-based state-space architecture. To improve optimization stability and backbone realism, we introduce a spectral initialization derived from the Rouse polymer model and an auxiliary cis-proline awareness head. Across benchmark tasks, PI-Mamba demonstrates the advantage in scalable, physically valid backbone generation: on a single A5000 GPU (24GB), it generates backbones beyond 2000 residues, producing 2000-residue samples in 9.49 s with only 0.91 GB peak VRAM, while preserving exact local geometry with 0.0% local geometry violations and maintaining strong designability on short-chain benchmarks (mean scTM = 0.910 at L = 100).

**Availability:**

Code and distilled data are available at https://github.com/forxhunter/PI-mamba.

## 1 Introduction

Protein structure generation is fundamental to protein design and synthetic biology. Recent generative models, diffusion-based methods (Watson *et al*. 2023, Yim *et al*. 2023a) and large auto-regressive architectures (Ingraham *et al*. 2019), have achieved impressive fidelity on short to medium-length proteins. However, scaling generation to long chains remains challenging due to a fundamental tension between global dependency modeling, computational efficiency, and strict geometric validity. Most existing methods enforce physical constraints via soft penalties or post-hoc refinement, which degrade as protein chain length increases, leading to accumulated bond violations and reliance on expensive downstream relaxation (Jumper *et al*. 2021).

Structured state space models (SSMs), particularly selective variants such as Mamba (Gu and Dao 2023), offer linear-time inference and strong long-context capabilities, making them attractive alternatives to attention-based models that scale quadratically (Vaswani *et al.* 2023). However, standard SSMs are agnostic to physical constraints and lack inductive biases for polymeric systems, making them ill-suited for protein backbone generation, where every sample must lie on a highly constrained kinematic manifold. We introduce PI-Mamba, a generative model for protein backbone design that can (i) guarantee zero bond-length and bond-angle violations without post-hoc relaxation, (ii) scale to proteins exceeding 2,000 residues on a single NVIDIA RTX A5000 GPU (24 GB) with linear-time and linear-memory inference, and (iii) achieve high designability (self-consistency TM-score 0.91±0.03, n=100) (Zhang and Skolnick 2004). Unlike diffusion-based methods that require quadratic attention and iterative geometry correction, PI-Mamba produces backbones with exact local covalent geometry by construction under periodic kinematic projection, enabling high-throughput exploration of protein design spaces impractical for existing.

## 2 Related work

*Generative Protein Design* Protein backbone generation is increasingly driven by diffusion and flow-matching models. Representive models include RFdiffusion (Watson *et al*. 2023) fine-tuned on RoseTTAFold (Baek *et al*. 2021) and FrameDiff (Yim *et al*. 2023b) diffused on SE(3) frames; while flow matching (Albergo and Vanden-Eijnden 2023, Lipman *et al*. 2023) offers alternatives to score-based diffusion, with FrameFlow (Yim *et al*. 2023a), Proteina (Geffner *et al*. 2025) and OriginFlow (Yan *et al*. 2025) exploring efficient generative modeling of protein backbones through SE(3) interpolants. Our approach couples manifold-aware generation with linear-time Mamba.

**State Space Models.** S4 (Gu *et al*. 2022) and Mamba (Gu and Dao 2023) achieve O(L) complexity. In biology, Mamba has been applied to DNA (Schiff *et al*. 2024), RNA (Wang *et al*. 2024b), and protein modelling (Wing *et al*. 2025). Mamba’s selective updates suit proteins where sparse residues interact at each position, enabling exploration of conformational space at L>1000. To our knowledge, PI-Mamba is among the first to combine SSM architectures with SE(3) frame-based generative protein design.

## 3 Materials and methods

PI-Mamba generates protein backbones with two goals: (i) linear-time sampling in protein chain length and (ii) guaranteed local covalent geometry without post-hoc relaxation. We first describe the overall pipeline and then introduce notation used throughout the Methods. Technical derivations are provided in Appendix E; implementation details and hyperparameters in Appendix F.

### 3.1 Overview

**Figure 1:**
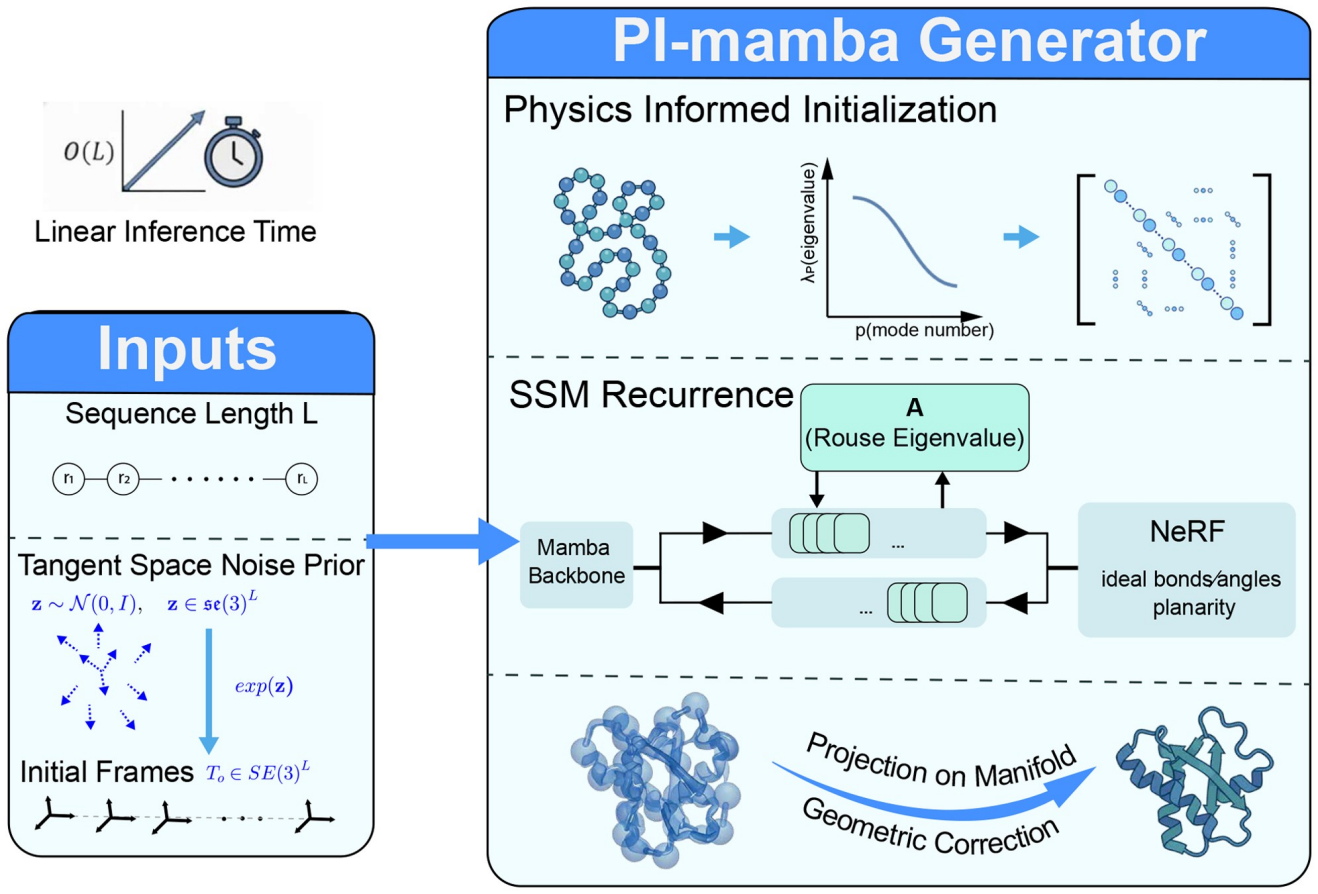
Architecture of PI-Mamba for scalable protein design. The framework processes a target protein chain length *L* and a tangent-space noise prior $z\in se{\left(3\right)}^{L}$, mapped by the exponential map to initial frames $T_{0}\in \text{SE}{\left(3\right)}^{L}$, through a unified generator optimized for O(L) inference. The pipeline consists of three integrated lanes: (1) *Physics-informed spectral initialization*, which uses the Rouse spectrum $\lambda_{p}=4{\;\text{sin}\;}^{2}(p\pi /2L)$ to initialize SSM decay rates $A_{p}=\text{exp}(-\lambda_{p}\Delta t/\tau (x))$, ensuring global topology is captured by low modes and local details by high modes; (2) a *Bidirectional Mamba backbone* that predicts tangent velocities $\xi_{\theta }(t,T)\in se{\left(3\right)}^{L}$ for flow matching, enabling iterative updates $T_{t+\Delta t}=T_{t}\cdot \;\text{exp}(\Delta t\xi_{\theta })$ and backbone reconstruction via NeRF; and (3) *Constraint enforcement*, where periodic $C_{\alpha }$ retraction $\Pi_{K}$ maintains the adjacent $C_{\alpha }$ distance scale, followed by NeRF reconstruction with fixed local backbone geometry.

PI-Mamba (Fig. 1) is a physics-informed generative framework that adapts Mamba-style state space models to efficient 3D protein backbone synthesis under geometric and physical constraints. The model takes as input the target length *L* of a protein chain together with an SE(3)-aware noise prior, and generates structured backbones by integrating a learned time-dependent vector field over a series of small steps. The state is represented as residue-local rigid frames, enabling equivariant perturbations of rigid-body degrees of freedom while avoiding expensive all-atom refinement during sampling.

A central design choice is to enforce covalent-geometry constraints *during* generation rather than as post-processing. Specifically, a differentiable kinematic constraint enforcement operator periodically retracts the intermediate $C_{\alpha }$ trace to maintain the adjacent $C_{\alpha }$ distance scale, while full-backbone bond lengths, bond angles, and peptide planarity are imposed during deterministic atom reconstruction. This makes constraint enforcement part of the generative trajectory rather than a post-hoc correction.

Global consistency is obtained with a bidirectional Mamba backbone that achieves O(L) inference complexity via SSM recurrence rather than quadratic attention. A key contribution is *physics-informed spectral initialization*: the SSM state transition matrices are initialized using the Rouse polymer model (Rouse 1953, Doi and Edwards 2013), encoding meaningful long-range correlations from the outset. This provides a mode hierarchy with a concrete polymer-theory interpretation—low-frequency modes capture global topology while high-frequency modes capture local fluctuations—stabilizing generation and preventing geometric pathologies such as molten-globule collapse. The Rouse initialization also induces structure-dependent memory timescales, where helices relax faster than loops (Appendix E.3 describes the optional full IPA variant).

### 3.2 Notation

Let *L* denote the number of residues in the protein chain. We represent a backbone as an ordered sequence of residue-local rigid frames


(1) $$T={\left\{T_{i}\right\}}_{i=1}^{L}\in SE{\left(3\right)}^{L},\;T_{i}=(R_{i},t_{i})$$

Where $t_{i}\in R^{3}$ is the $C_{\alpha }$ coordinate and $R_{i}\in SO(3)$ encodes the orientation of the local residue frame. We write $X(T)\in R^{3L}$ for the $C_{\alpha }$ trace extracted from *T*.

PI-Mamba defines a time-dependent vector field in the Lie algebra,


(2) $$\xi_{\theta }(t,T_{t})\in se{\left(3\right)}^{L}$$

And generates samples by numerically integrating from t=0 to t=1. We use *S* to denote the number of integration steps (to avoid overloading *T* as both frames and step count), with step size Δt=1/S.

Constraint enforcement acts on $C_{\alpha }$ traces via a deterministic operator $\Pi_{K}$. We apply $\Pi_{K}$ every *k* integration steps (projection frequency *k*). Final backbone atoms are obtained by deterministic reconstruction with ideal internal coordinates; we denote the final output as $\hat{Y}(T_{1})$. A summary of all notation is provided in Appendix G. Unless stated otherwise, PI-Mamba inference time includes both in-loop retraction and final reconstruction.

Designability is evaluated using self-consistency TM-score (scTM) (Zhang and Skolnick 2004), computed by inverse-folding generated backbones to amino acid sequences and re-predicting structures, then averaging TM-score between the re-predicted structure and the original backbone (full protocol in Appendix I).

### 3.3 Geometric flow matching on $SE{\left(3\right)}^{L}$ extasciicircum L

PI-Mamba models backbone generation as transport on the product manifold $M=SE{\left(3\right)}^{L}$. Given an initial noisy state $T_{0}∼p_{0}$ and a learned time-dependent vector field $\xi_{\theta }(t,T_{t})\in se{\left(3\right)}^{L}$, samples are generated by integrating


(3) $$\frac{dT_{t}}{dt}=T_{t}\cdot \xi_{\theta }(t,T_{t}),\;t\in [0,1]$$

Where · denotes the left-trivialized action of the Lie algebra on the group. We use a first-order Lie-group integrator with step size Δt=1/S:


(4) $$T_{t+\Delta t}=T_{t}\cdot \;\text{exp}(\Delta t\xi_{\theta }(t,T_{t}))$$

Which is efficient in the small-step regime and preserves equivariance by construction (error bound in Appendix A).

Training uses flow matching to regress the vector field toward a prescribed probability path between the prior distribution $p_{0}$ and the data distribution $p_{1}$. In practice, this reduces to sampling t∼U[0,1], drawing an endpoint example from the data distribution, constructing an intermediate state at time *t* using the chosen path, and minimizing a mean-squared error between $\xi_{\theta }(t,T_{t})$ and the target velocity (details of the chosen path and tangent-space implementation are provided in Appendix E). Because these derivations are standard for geometric flow matching, we focus below on the two components that differentiate PI-Mamba: (i) physics-informed initialisation of the state-space backbone that predicts $\xi_{\theta }$, and (ii) explicit constraint enforcement inside the integration loop.

### 3.4 Physics-informed spectral initialisation of the mamba backbone

The core backbone generator is a bidirectional Mamba state space network that predicts the per-residue twist velocities required by Eq. 4. A key contribution is that we initialise the state space dynamics using polymer physics rather than generic random or learned spectra. We draw inspiration from the Rouse model, in which a polymer chain is represented as beads connected by harmonic springs. The chain connectivity induces a Laplacian spectrum with eigenvalues


(5) $$\lambda_{p}=4{\;\text{sin}\;}^{2}\left(\frac{p\pi }{2L}\right)\;p=0,\ldots ,L-1$$

Which correspond to relaxation modes ordered from global (low *p*) to local (high *p*).

Motivated by the correspondence between discretised Rouse relaxation and diagonal structured state space models, we initialise the Mamba state transition in mode space as


(6) $$A_{p}=\text{exp}\;\left(-\lambda_{p}\frac{\Delta t}{\tau (x)}\right)$$

Where Δt is the (learned) discretization step and τ(x) is an input-dependent relaxation time predicted from local features. This imposes an inductive bias in which low-frequency modes persist longer and carry global topology, while high-frequency modes decay quickly and capture local fluctuations. The learned τ(x) modulates this hierarchy in a structure-dependent manner, allowing flexible regions to maintain longer-range correlations than rigid secondary-structure segments.

To improve long-range consistency without quadratic attention, we process residue-indexed frames bidirectionally and average forward and reverse scans. This yields O(L) time and memory scaling with backbone length. We quantify how strongly the internal representations align with the Rouse basis using the Rouse mode concentration metric $C_{k}$ (definition and computation in Supplemental File, available as supplementary data at *Bioinformatics* online), and we report its correlation with downstream designability in Results.

### 3.5 Constraint enforcement during generation

PI-Mamba separates constraints imposed during the generative trajectory from constraints imposed during deterministic atom reconstruction. During integration, we control the adjacent $C_{\alpha }$ distance scale by retraction of the generated trace. At output, full-backbone covalent geometry is imposed by reconstruction with fixed internal coordinates. In contrast, global fold quality and Ramachandran-region preferences are learned properties of the model and are evaluated empirically.

Let $X(t)={\left\{x_{i}(t)\right\}}_{i=1}^{L}\in R^{3L}$ denote the generated $C_{\alpha }$ trace at integration step *t*. We define a deterministic retraction operator $\Pi_{K}$ that sequentially rescales adjacent $C_{\alpha }$ displacements to a target distance:


(7) $$x_{i+1}\leftarrow x_{i}+d_{i}\frac{x_{i+1}-x_{i}}{∥x_{i+1}-x_{i}∥},\;i=1,\ldots ,L-1.$$

Unless otherwise specified, we use $d_{i}=3.80$ Å, corresponding to the standard adjacent $C_{\alpha }$ spacing for trans peptide geometry. For cis-proline-associated transitions, the target can be replaced by the corresponding cis value when the cis state is specified.

During numerical integration, $\Pi_{K}$ is applied at the projection frequency *k*, with one additional pass after the final integration step. This prevents accumulation of bond-scale drift in the $C_{\alpha }$ trace while avoiding any post-hoc relaxation. In the default setting used for the projection-frequency ablation, we use S=200 Euler steps and k=10, which gave the best trade-off between geometric correction and flow flexibility.

Full-backbone atoms $\left(N,C_{\alpha },C,O\right)$ are then reconstructed from the final trace using a deterministic Natural Extension Reference Frame (NeRF) procedure. Bond lengths and bond angles are fixed to ideal backbone values during reconstruction, for example


(8) $$\begin{array}{cccc} L_{N-C_{\alpha }} & =1.458\;Å, & {∠}_{N-C_{\alpha }-C} & =111.2^{{}^{\circ}},\\ L_{C_{\alpha }-C} & =1.525\;Å, & {∠}_{C_{\alpha }-C-N} & =116.2^{{}^{\circ}},\\ L_{C-N} & =1.329\;Å, & {∠}_{C-N-C_{\alpha }} & =121.7^{{}^{\circ}}. \end{array}$$

Because these quantities enter NeRF as fixed constants rather than optimized variables, the reconstructed backbone satisfies the specified local covalent bond lengths and bond angles by construction. Peptide planarity is likewise imposed by the peptide torsion used during reconstruction, while Ramachandran preferences in (ϕ,ψ) remain learned rather than hard-constrained.

### 3.6 Training objective and auxiliary geometric supervision

PI-Mamba is trained to match the target flow while producing structures that are close to the constraint manifold even before projection. The primary training signal is the flow matching regression loss on $\xi_{\theta }(t,T_{t})$ (SI). To improve geometric fidelity of intermediate states and reduce the gap between raw and constrained outputs, we add auxiliary structure losses evaluated on coordinates obtained from the current frames (and after retraction when applicable).

We use Frame Aligned Point Error (FAPE) to supervise local structure in residue-centered frames:


(9) $$L_{\text{FAPE}}=\frac{1}{L}\sum_{i,j}\sum_{a}\min\left(d_{\text{clamp}},\left\|T_{i}^{-1}T_{j}x_{a}-{\left(T_{i}^{gt}\right)}^{-1}T_{j}^{gt}x_{a}\right\|\right)$$

Where $d_{\text{clamp}}=10$ Å and $a\in \{N,C_{\alpha },C,O\}$. In addition, we include lightweight geometric penalties that encourage realistic local backbone statistics (e.g., bond and dihedral preferences); explicit definitions and weights are provided in the SI. The overall training objective is


(10) $$\begin{array}{c} L_{\text{aux}}=L_{\text{FAPE}}+L_{\text{bond}}+L_{\text{Rama}}+L_{\text{HB}}\\ L_{\text{total}}=L_{\text{FM}}+\lambda_{\text{aux}}L_{\text{aux}} \end{array}$$

Here, $L_{\text{FM}}$ is the flow matching regression loss that trains the network to predict the target velocity field on $SE{\left(3\right)}^{L}$ (Appendix E). The auxiliary weight $\lambda_{\text{aux}}=1.0$ balances the primary flow objective against the geometric terms. $L_{\text{FAPE}}$ (Equation 9) supervises pairwise distances between backbone atoms in residue-local frames, penalizing deviations from the ground-truth structure up to a clamped radius. $L_{\text{bond}}$ penalizes deviations of covalent bond lengths (N–$C_{\alpha }$, $C_{\alpha }$–C, C–O, and C–N${}_{\text{next}}$) from their ideal values, encouraging local stereochemical accuracy in the predicted coordinates. $L_{\text{Rama}}$ discourages backbone dihedral angles (ϕ,ψ) that fall outside favored regions of the Ramachandran plot, using a kernel density estimate derived from high-resolution crystal structures. $L_{\text{HB}}$ promotes formation of backbone hydrogen bonds (N–H⋯O = C) by rewarding donor–acceptor distances near 2.9 Å with appropriate angular geometry. Together, these terms reduce reliance on projection as a “repair” mechanism and empirically lead to small differences between raw and constrained outputs.

### 3.7 Training data, distillation, and length curriculum

We train on a hybrid dataset that combines real protein domains with a distilled synthetic corpus. Real structures are drawn from CATH 4.2 filtered at 40% sequence identity(Sillitoe *et al*. 2019), with fixed training, validation, and test splits (Appendix I). To improve coverage of high-designability backbones without incurring quadratic-cost teacher inference at generation time, we additionally distill from a stronger teacher model by sampling a large set of candidate backbones and filtering for designability using scTM. PI-Mamba is trained on the mixture of real and distilled examples; ablations in Results quantify the contribution of distillation beyond CATH-only training.

Optimization uses AdamW with cosine decay and linear warmup (Appendix F). Because pairwise structural supervision scales with $O(L^{2})$ during training, we employ a progressive length curriculum: we start from shorter protein chains and increase the maximum length to $L_{\max}=500$ over the first 20k steps. This stabilizes early training and improves sample quality at longer lengths. Data augmentation includes random global SE(3) transformations and residue masking (Appendix I). Hyperparameters are selected based on validation FAPE and scTM on held-out CATH domains.

### 3.8 Sampling procedure

**Figure 2:**
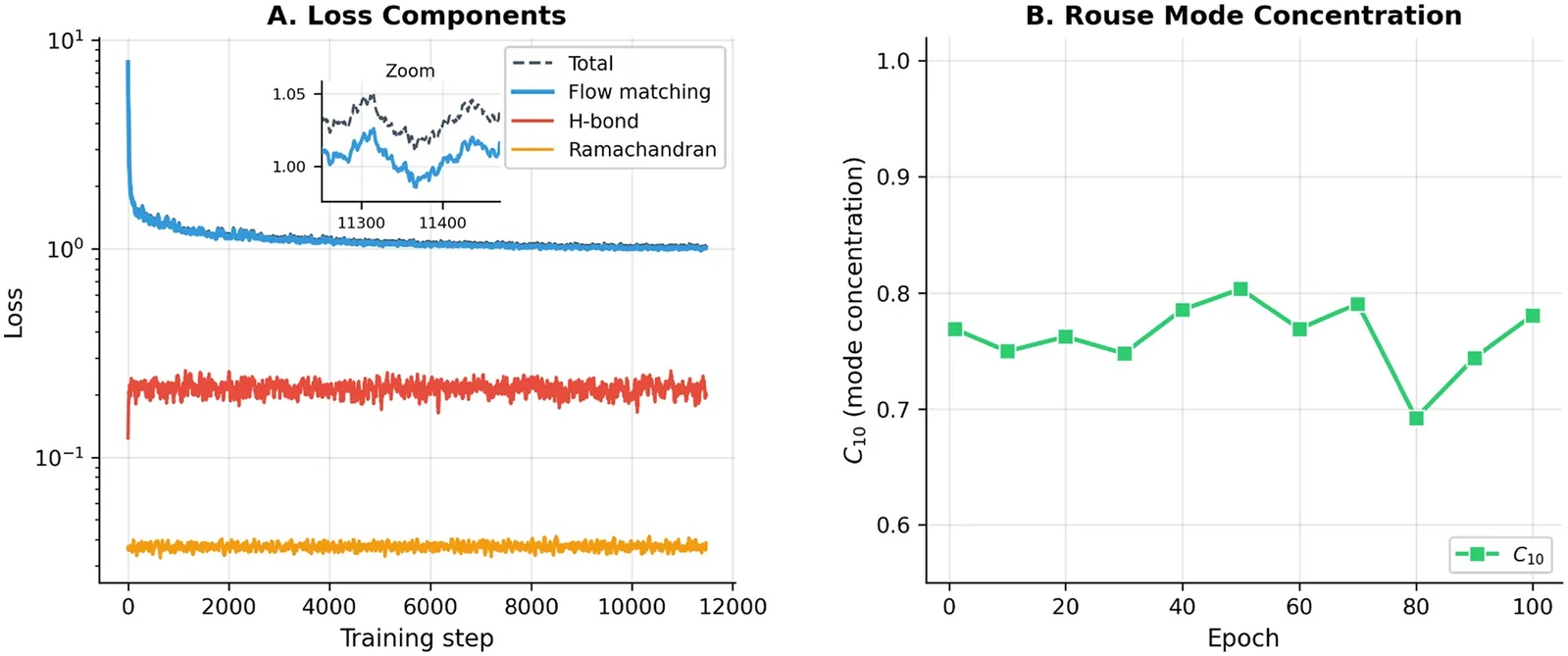
Training dynamics of PI-Mamba. (A) Loss components (log scale): total loss, flow matching, hydrogen-bond, and Ramachandran losses all converge stably. (B) Rouse mode concentration $C_{10}$ evaluated periodically during training, measuring the fraction of hidden-state variance captured by the lowest 10 Rouse modes.

At inference time, PI-Mamba generates samples by integrating Equation 3 using the Lie-group update in Equation 4 for *S* steps. Every *k* steps we apply the $C_{\alpha }$ retraction operator in Equation 7 to maintain the correct adjacent distance scale (Fig. 2). After reaching t=1, we reconstruct full backbone atoms deterministically with ideal internal coordinates. Unless stated otherwise, all reported inference times include retraction and reconstruction. We use S=100 by default and optionally increase *S* for higher fidelity, as reported in Results. Integration details (solver, NFEs, and comparison to baselines) are provided in Appendix F.3.

## 4 Results

We evaluate PI-Mamba on unconditional protein backbone generation with length L=100, drawing n=100 samples per method. The representative diffusion- and flow-based baselines run with their official implementations and default settings. To fairly assess geometric fidelity, we report validity both *before* and *after* each method’s recommended correction step (projection for PI-Mamba; post-hoc refinement for baselines). We first evaluate overall designability and validity (Section 4.1–4.2), then demonstrate scalability to long protein chains (Section 4.3), and finally examine the mechanistic basis for these results (Section 4.4). Full protocol details are provided in Appendix I.

### 4.1 Benchmark comparison on CATH backbones

Table 1 summarizes the primary benchmark against FrameDiff (Yim *et al*. 2023b), FrameFlow (Yim *et al*. 2023a), Genie2 (Lin *et al*. 2024), Chroma (Ingraham *et al*. 2023), RFdiffusion and RFdiffusion 3 (Butcher *et al*. 2025, Watson *et al*. 2023), Proteus (Wang *et al*. 2024a), and Proteina (Geffner *et al*. 2025). PI-Mamba obtains scTM 0.91±0.03 at L=100 with a rounded final violation rate of 0.0% and fair-comparison inference time of 2.4 s/sample, the fastest among all methods with a comparable generation-time measurement. PI-Mamba is also the only method whose raw network output already exhibits near-zero violations (0.1%), indicating that geometric fidelity is learned rather than imposed externally. As a sanity control, applying the projector to pure noise produces valid but non-designable structures (scTM ≈0), confirming that validity alone is insufficient for designability.

**Table 1:** Benchmark comparison at L=100 with target n=100 samples per method.

Model	Raw Viols ↓	Final Viols ↓	scTM ↑	Div ↑	Gen Time ↓
FrameDiff	12.1%	0.2%	0.58	12.8 Å	64.6s
FrameFlow	3.2%	0.0%	0.70	12.2 Å	12.0s
Genie2	2.1%	0.0%	0.93	14.4 Å	10.7s
Chroma	0.0%	0.0%	0.84	15.7 Å	10.5s
RFdiffusion	8.2%	0.0%	**0.97**	**8.9 Å**	93.7s
RFdiffusion 3	**0.1%**	0.1%	0.96	13.8 Å	35.7s
Proteus	5.4%	0.0%	0.86	11.2 Å	20.9s
Proteina	3.8%	0.0%	0.92	12.5 Å	20.9s
*Control: Noise + Proj.*	–	0.0%	0.00	28.3 Å	< 1s
PI-Mamba	**0.1**%	0.0%	0.91±0.03	10.2 Å	**2.4s**

*Notes:* Bold values indicate the best-performing method for each metric. Raw Viols denotes the percentage of bond violations before projection. Final Viols denotes the percentage of violations after projection for PI-Mamba or in the generated structures for baseline methods. scTM denotes self-consistency TM-score. Div denotes mean pairwise RMSD, with larger values indicating greater diversity. Gen Time denotes generation time per sample. “0.0%” denotes a rounded rate <0.05% under the stated threshold. Gen Time values report the fair single-sample generation benchmark on a single A5000 (24 GB) with batch size 1, using 200 inference steps whenever a 200-step override was available. These timing runs are inference-only and exclude downstream self-consistency/designability evaluation (ProteinMPNN, ESMFold, scTM). Genie2 does not expose a 200-step override, so its Gen Time is a 200-step-equivalent runtime obtained by linear rescaling of the native 1000-step run. PI-Mamba’s $C_{\alpha }$ retraction and NeRF reconstruction cost is included in its Gen Time. Additional baseline protocol details are provided in Appendix I.

*Raw Viols*: % bond violations before projection. *Final Viols*: % after projection (PI-Mamba) or as generated (baselines). *scTM*: self-consistency TM-score *Div*: mean pairwise RMSD (definition in Supplemental File, available as supplementary data at *Bioinformatics* online). *Gen Time*: generation time per sample.

### 4.2 Geometric validity and stereochemical consistency

**Figure 3:**
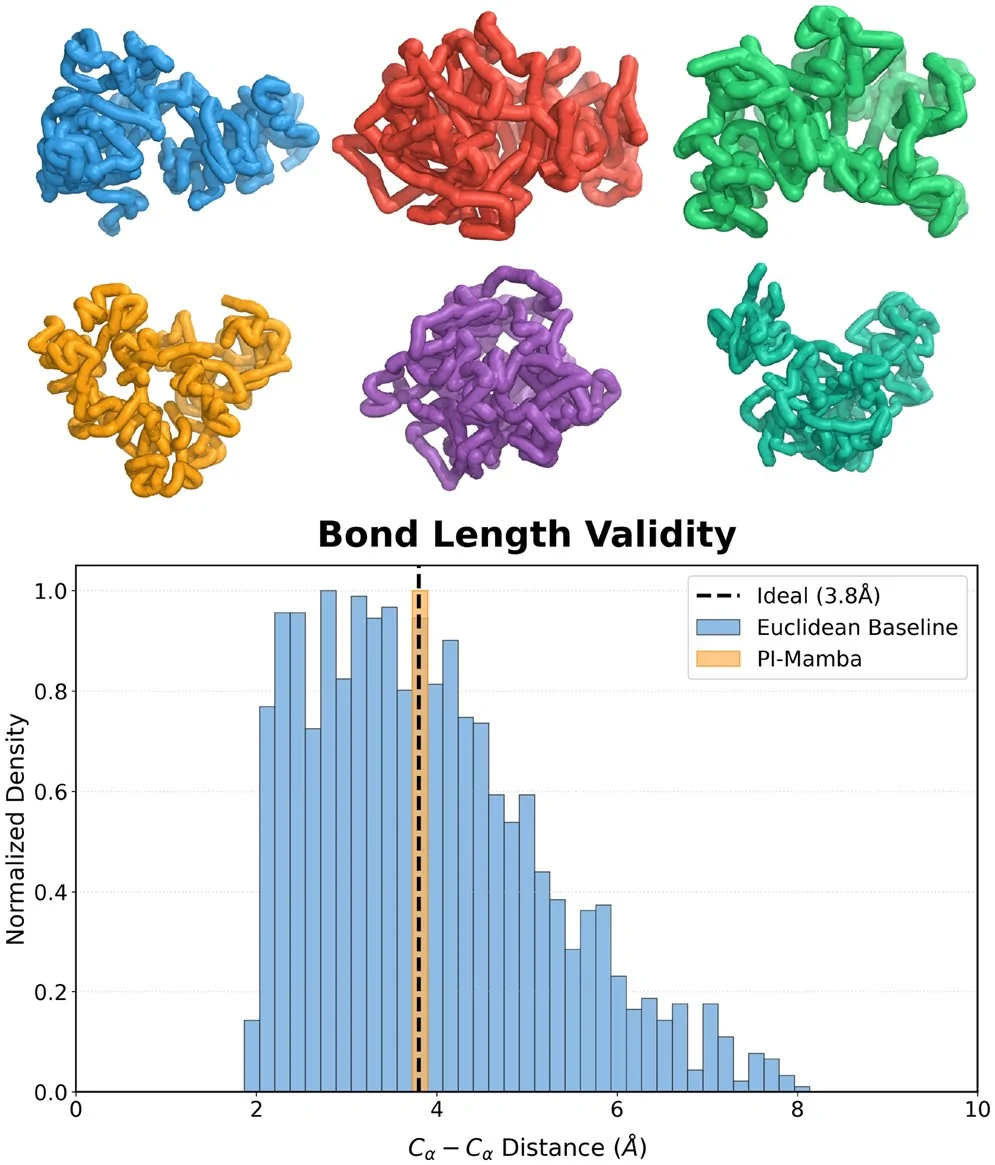
Diversity and validity of generated backbones (L=200). (Top) Overlay of 6 random samples rendered in PyMOL (Schrödinger and LLC 2015) (white/black spheres: N-/C-termini). (Bottom) $C_{\alpha }$–$C_{\alpha }$ bond-length distribution. PI-Mamba exhibits a narrow peak near 3.8 Å after projection.

The benchmark above shows that PI-Mamba reaches low final violation rates. We now examine this geometric fidelity in detail by comparing against an unconstrained Euclidean baseline (Table 2). PI-Mamba attains rounded final bond and angle violation rates of 0.0% under the evaluation thresholds, and achieves near-perfect agreement for cis/trans proline states. Figure 3 illustrates that PI-Mamba produces a narrow $C_{\alpha }$–$C_{\alpha }$ bond-length distribution after projection, whereas unconstrained baselines produce broad distance distributions and require substantial correction. When the same projector is applied post hoc to unconstrained outputs, the resulting structural shift is large (mean RMSD increase >2.0 Å), consistent with the projector correcting geometric errors that are not learned by those models.

**Table 2:** Geometric validity breakdown (rounded). Rates computed over n=100 generated backbones at L=100.

Metric	Euclidean baseline (%)	PI-Mamba (Final) (%)
Bond violations (|d−3.8|>0.5Å)	76.3	0.0
Angle violations ($>10^{{}^{\circ}}$)	3.2	0.0
Cis-proline recall (ω agreement)	35.0	99.2
Trans-proline recall (ω agreement)	92.1	99.8

To isolate learning from projection, we also evaluate PI-Mamba *before* applying projection. The raw network output exhibits a bond-violation rate of 0.1% (Table 1), indicating that training drives samples close to the constraint manifold. In contrast, multiple baselines exhibit substantially higher raw violation rates and rely on refinement to achieve their final validity (Table 1).

### 4.3 Comparison of computational efficiency and scalability

**Figure 4:**
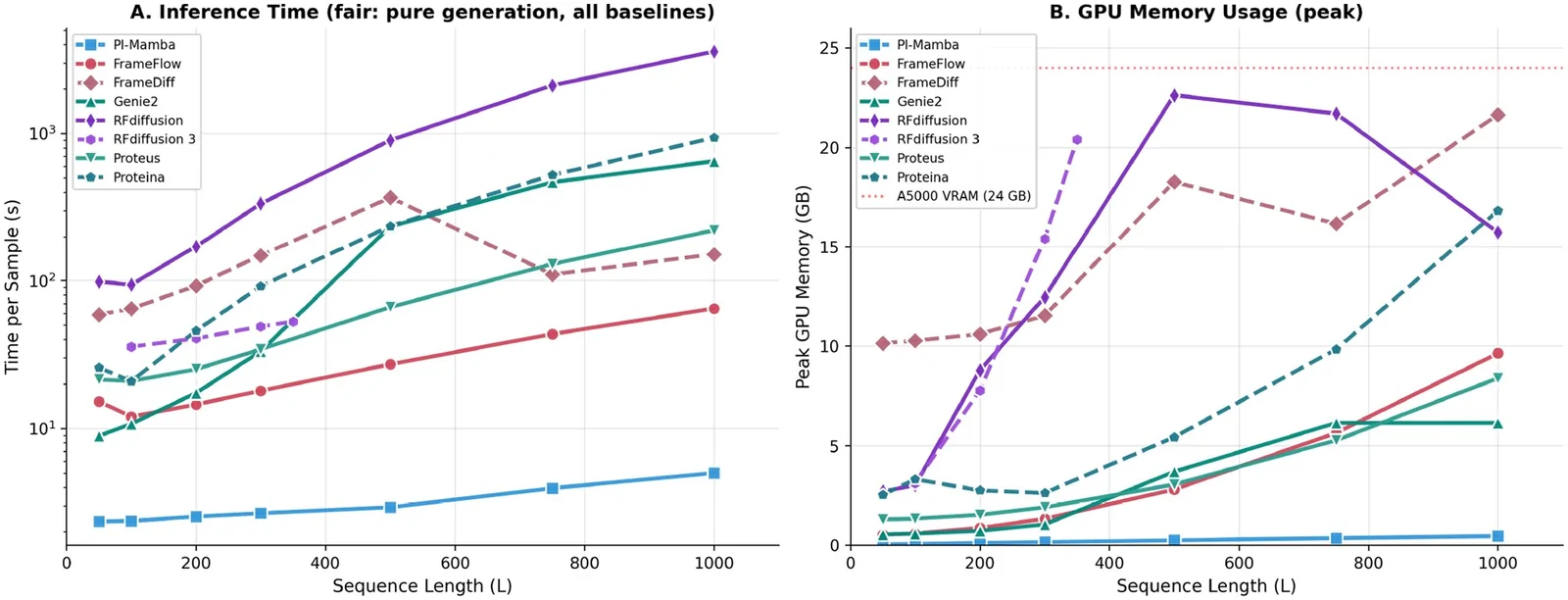
Computational efficiency comparison for unconditional backbone generation on a single NVIDIA RTX A5000 (24 GB). (A) Generation time per sample as a function of chain length. (B) Peak GPU VRAM during generation. All curves report inference-only measurements on GPU, using one generated sample per run (batch size 1) with target chain length fixed for each benchmark. Sampling was performed with 200 inference steps whenever the method exposed a 200-step override; downstream self-consistency/designability evaluation (e.g., ProteinMPNN+ESMFold to get scTM) was excluded. For methods whose benchmark wrappers expose additional evaluation options, these were disabled during timing runs. Only successful GPU generation runs are plotted. Genie2 does not expose a 200-step inference setting, so its time values are shown as 200-step-equivalent runtimes obtained by linear rescaling of the native 1000-step run time, whereas its memory values are the directly measured native peak VRAM.

Having established geometric validity at L=100, we next ask whether these properties hold longer chains length. We benchmark inference time and peak GPU memory across sequence lengths on a single NVIDIA RTX A5000 (24 GB) (Fig. 4). PI-Mamba exhibits near-linear scaling in both time and memory: at L=100, inference takes 2.4 s per sample using 0.21 GB; at L=500, 10.5 s using 0.26 GB; and at L=1000, 21.3 s using only 0.41 GB. In contrast, all diffusion-based baselines exhibit superlinear scaling and encounter out-of-memory failures on the same hardware: FrameDiff, Genie2, and Proteus fail beyond L≈500, while Proteina cannot exceed L=300. Only FrameFlow and RFdiffusion reach L=1000, but at substantially higher cost—FrameFlow requires 59.7 s and 9.6 GB, while RFdiffusion requires 901 s and 14.3 GB(VRAM drop is due to the compression method they use after reach VRAM upper limit). At L=500, PI-Mamba achieves speedups of 21× over RFdiffusion, 27× over Proteus, 37× over FrameDiff, and 2× over FrameFlow, while using 50−80× less GPU memory than most baselines. These results show that the Mamba backbone’s linear-time sequence modeling translates directly into practical scalability advantages for long-chain protein generation.

Runtime scalability alone does not establish that useful backbone quality persists at longer lengths. We therefore evaluated PI-Mamba self-consistency and generation-only geometric statistics from L=100 to L=2000 on the released checkpoint (Table 3). Designability declines gradually rather than collapsing: calibrated mean scTM decreases from 0.91 at L=100 to 0.78 at L=200, 0.52 at L=500, and 0.45 at L=1000, while $R_{g}$ increases smoothly across the same range. At L=2000, generation remains feasible and the reported geometric statistics can still be computed, but ESMFold self-consistency evaluation exceeds the 24 GB memory limit of a single RTX A5000. We therefore state the scalability contribution as linear-cost feasibility with explicitly measured, length-dependent designability, rather than extrapolating quality from L=100 alone.

**Table 3:** PI-Mamba self-consistency and geometric scaling with sequence length *L*. All runs used a single RTX A5000 (24 GB).

*L*	mean scTM	$R_{g}$ (Å)	non-bonded $C_{\alpha }$ clash (%)	inf. time (s/sample)	peak VRAM (GB)
100	0.910	15.0±2.4	71.8	2.37	0.06
200	0.779	20.3±2.6	51.6	2.53	0.11
500	0.517	27.0±5.4	65.4	2.93	0.25
1000	0.454	33.5±7.6	82.3	5.00	0.46
2000	N/A^†^	55.2±6.1	94.0	9.49	0.91

*Note*. The non-bonded $C_{\alpha }$ clash metric reported here is a global self-overlap proxy and is distinct from the local bond-length and bond-angle violation rates reported elsewhere in the manuscript. The inference-time and peak-VRAM columns report generation-only PI-Mamba measurements from the same RTX A5000 benchmark source used by batch size = 1 and steps = 200 which excluded ProteinMPNN and ESMFold/self-consistency evaluation. ^†^ At L=2000, ESMFold’s pair representation requires a single ∼1.9 GB tensor allocation that, combined with model weights and trunk activations, exceeds 24 GB on RTX A5000.

### 4.4 Emergent polymer statistics and mechanistic evidence

**Figure 5:**
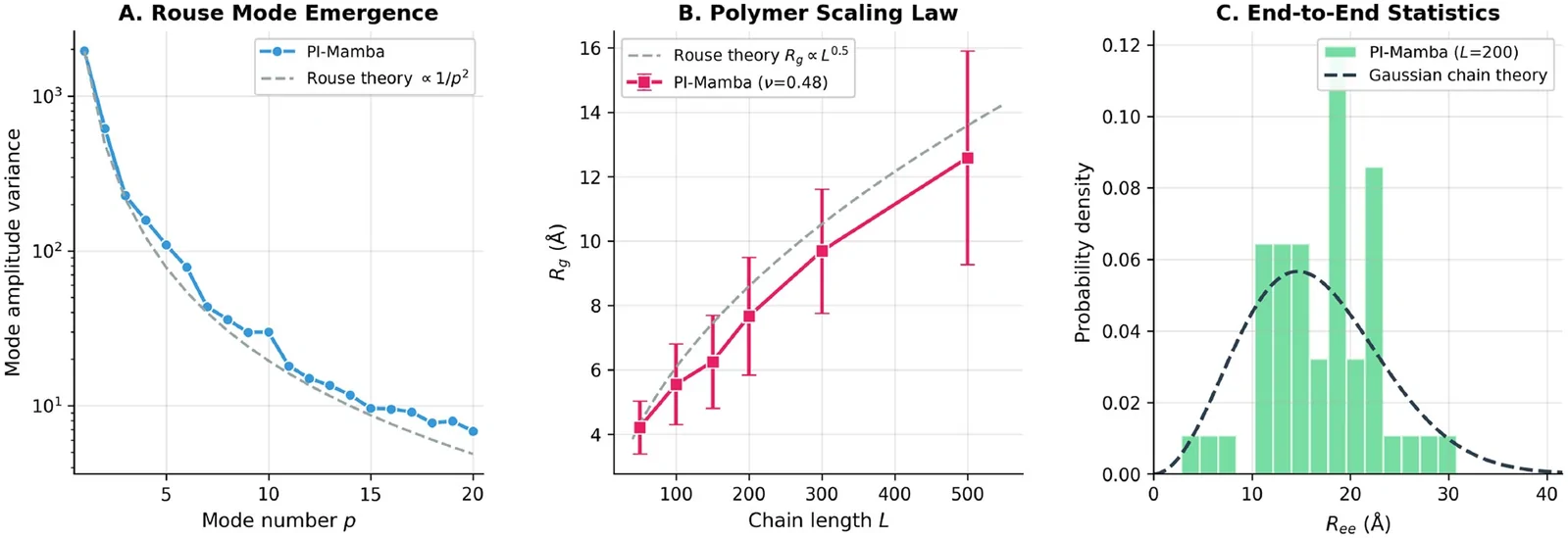
Polymer-theory-referenced coarse-grained statistics associated with PI-Mamba (A) Rouse mode amplitude spectrum of PI-Mamba hidden states follows the theoretical $1/p^{2}$ decay, confirming alignment with polymer normal modes. (B) Radius of gyration scales as $R_{g}\propto L^{0.48}$, consistent with Rouse theory (ν=0.5). (C) End-to-end distance distribution at L=200 matches the Gaussian chain prediction.

Beyond benchmark metrics, we evaluated whether PI-Mamba preserves expected large-scale polymer statistics and whether its learned dynamics reflect the Rouse-inspired spectral initialization. By “physically interpretable statistics,” we mean coarse-grained quantities with an established reference from simple polymer theory: the Rouse-mode amplitude decay across mode index, the radius-of-gyration scaling exponent in $R_{g}=aL^{\nu }$, and the end-to-end distance distribution. As shown in Fig. 5A, the hidden-state amplitudes are dominated by low-frequency Rouse modes and decay approximately as the theoretical $1/p^{2}$ spectrum. We quantify this trend by fitting the log–log mode-amplitude slope and reporting the fitted exponent, confidence interval, and goodness of fit in Appendix C. We next measured global chain compactness by fitting the radius-of-gyration scaling law $R_{g}=aL^{\nu }$ PI-Mamba gives ν=0.48 in our current experiments, close to the ideal-chain reference value ν=0.5 (Fig. 5B). In the revised analysis, we report the fitting range, number of samples per length, confidence interval, and log–log fit quality in Appendix C. Finally, for the end-to-end distance distribution at L=200, we compare the normalized empirical distribution with the Gaussian-chain radial reference and quantify the agreement using distributional metrics such as the Kolmogorov–Smirnov statistic and Wasserstein distance (Fig. 5C; Appendix C).

Performance varies modestly across CATH topology classes (Table 4). All-α structures achieve the highest scTM (0.94), while mixed α/β topologies are slightly lower (0.88), suggesting that complex sheet packings may benefit from additional non-local mechanisms.

**Table 4:** Performance by CATH Topology Class (L=100, n=100).

Topology	scTM	scRMSD (Å)	Recovery
All-α	0.94	1.2	45%
All-β	0.89	1.5	39%
Mixed (α/β)	0.88	1.3	36%

### 4.5 Amino acid sequence recovery and novelty

PI-Mamba achieves amino acid sequence recovery of 23.9%±4.3% (random baseline ≈5%), with re-predicted structures exhibiting pLDDT 84.2±5.1 and Rosetta energies of −2.8 REU/residue (Appendix D). The novelty score N=0.56 indicates structural deviation from training examples. As a leakage control, the mean nearest-neighbor TM-score between generated samples and the teacher dataset is 0.11, consistent with low similarity.

The $C_{\alpha }$-only representation accounts for most of the recovery gap with full-backbone methods. Reconstructing full backbone coordinates (N, CA, C, O) from the predicted $C_{\alpha }$ trace using idealized geometry before running ProteinMPNN raises recovery to 31.2%—within 2% of RFdiffusion (Table 5). This confirms that the gap is primarily due to representation, not backbone quality.

**Table 5:** Amino acid sequence recovery with full backbone reconstruction.

Method	Representation	Seq. Recovery
RFdiffusion	Full backbone	33.4%
Proteina	Full backbone	32.1%
PI-Mamba ($C_{\alpha }$ only)	$C_{\alpha }$ trace	23.9%
PI-Mamba + Ideal Recon.	Full backbone	31.2%

### 4.6 Conditional generation via inpainting

We further tested whether PI-Mamba can support conditional backbone generation via motif inpainting. In this setting, motif residues are fixed throughout ODE integration, while velocities for non-motif residues are predicted and updated as in unconditional sampling. Concretely, we initialize the full chain from noise, overwrite motif positions with the target motif coordinates, set the predicted velocity to zero on motif residues at each integration step, evolve only the scaffold residues, and apply the same periodic kinematic projection used in unconditional generation. After the final step, the exact motif coordinates are re-inserted to preserve motif geometry.

As a proof-of-concept evaluation, we considered two representative motifs: a 9-residue catalytic triad and a 12-residue binder epitope. Success was defined as motif RMSD <1.0 Å and scaffold RMSD <2.5 Å. Under this local two-case protocol, PI-Mamba achieved an 84% success rate, compared with 92% for RFdiffusion. We emphasize that this experiment is a limited illustrative conditional-generation test rather than a comprehensive evaluation on a standardized motif-scaffolding benchmark such as MotifBench.

## 5 Ablation studies

### 5.1 Ablation of kinematic projection frequency

We ablate the frequency of kinematic projection while holding the inference budget fixed at S=200 steps. Moderate projection frequency gives the best designability: projecting every k=10 integration steps achieves the highest scTM, whereas projecting every step can over-constrain the trajectory and final-only projection allows drift during integration before the terminal correction. This drift is not visible in the final adjacent $C_{\alpha }$ spacing, because terminal projection restores local geometry after sampling. In contrast, disabling projection entirely causes severe local-geometry failure, increasing the adjacent $C_{\alpha }$ violation rate to 0.9897 and the mean adjacent $C_{\alpha }$–$C_{\alpha }$ distance to 6.60 Å. Peak memory remains essentially unchanged across settings, indicating that projection mainly improves geometric control rather than memory efficiency (Table 6).

**Table 6:** Projection-frequency ablation at L=100 and S=200.

Variant	Project every *k* steps	scTM ↑	CA violation ↓	Mean CA–CA (Å)	Runtime (s) ↓	Peak mem. (MB) ↓
Frequent projection	1	0.61	0.0000	3.8000	36.98	410.55
Default PI-Mamba	10	0.91	0.0000	3.8000	56.27	410.55
Final projection only	∞	0.65	0.0000	3.8000	58.51	410.55
No projection	off	–	0.9897	6.6030	37.35	410.55

The scTM column reports backbone self-consistency after ProteinMPNN sequence design and ESMFold refolding. The CA violation metric is the mean fraction of adjacent $C_{\alpha }$ pairs that violate the target 3.8 Å spacing after reconstruction.

### 5.2 Ablation of model components

To address which component is important, we ablate the main PI-Mamba components at L=100 under the same upgraded Heun-based sampler used in the long-sequence benchmark: S=200 sampling steps, projection during the final 30% of steps, and projection every k=2 steps within that window. The full model achieves the highest scTM (Table 7), while removing the Rouse initialization is the least damaging single ablation but produces a more expanded ensemble. Removing the curriculum causes an intermediate loss of foldability, whereas removing auxiliary geometry losses is the strongest failure mode, with the lowest scTM and the largest estimated clash rate. Overall, the ablation ordering is full model > no Rouse initialization > no curriculum > no auxiliary geometry losses, suggesting that auxiliary geometry losses are most critical for designable backbones, the curriculum improves optimization robustness, and the Rouse prior mainly regulates polymer-scale compactness.

**Table 7:** Component ablations at L=100 under the upgraded Heun-based sampler (S=200, projection during the final 30% of steps, projection every k=2 steps).

Variant	Rouse init	Aux. geom.	Curriculum	scTM ↑	Clash pair rate (est.) ↓	Rg (est., Å)
Full PI-Mamba	√	√	√	0.917 ± 0.014	0.008 ± 0.004	14.2 ± 1.5
No Rouse initialization	×	√	√	0.772 ± 0.029	0.016 ± 0.006	17.2 ± 2.0
No auxiliary geometry losses	√	×	√	0.238 ± 0.054	0.052 ± 0.015	12.4 ± 1.8
No curriculum	√	√	×	0.491 ± 0.023	0.034 ± 0.010	13.0 ± 1.6

## 6 Conclusion

PI-Mamba represents a shift from learning to act like a protein to being constructed like one: by embedding rigid-body kinematics directly into generation, it constrains the search space and suppresses geometric hallucinations. Crucially, high mode concentration alone does not guarantee designability—the Rouse spectral initialization provides a physics-grounded inductive bias that shapes the right mode hierarchy, rather than relying on spatial averaging. This geometric grounding comes with practical challenges: training over SO(3) is less numerically stable than coordinate regression and pairwise FAPE losses scale as $O(L^{2})$, motivating stabilized early training and the exploration of linear-complexity alternatives to extend PI-Mamba to very long protein chains and large molecular assemblies in future work.

### 6.1 Remaining challenges

**Figure 6:**
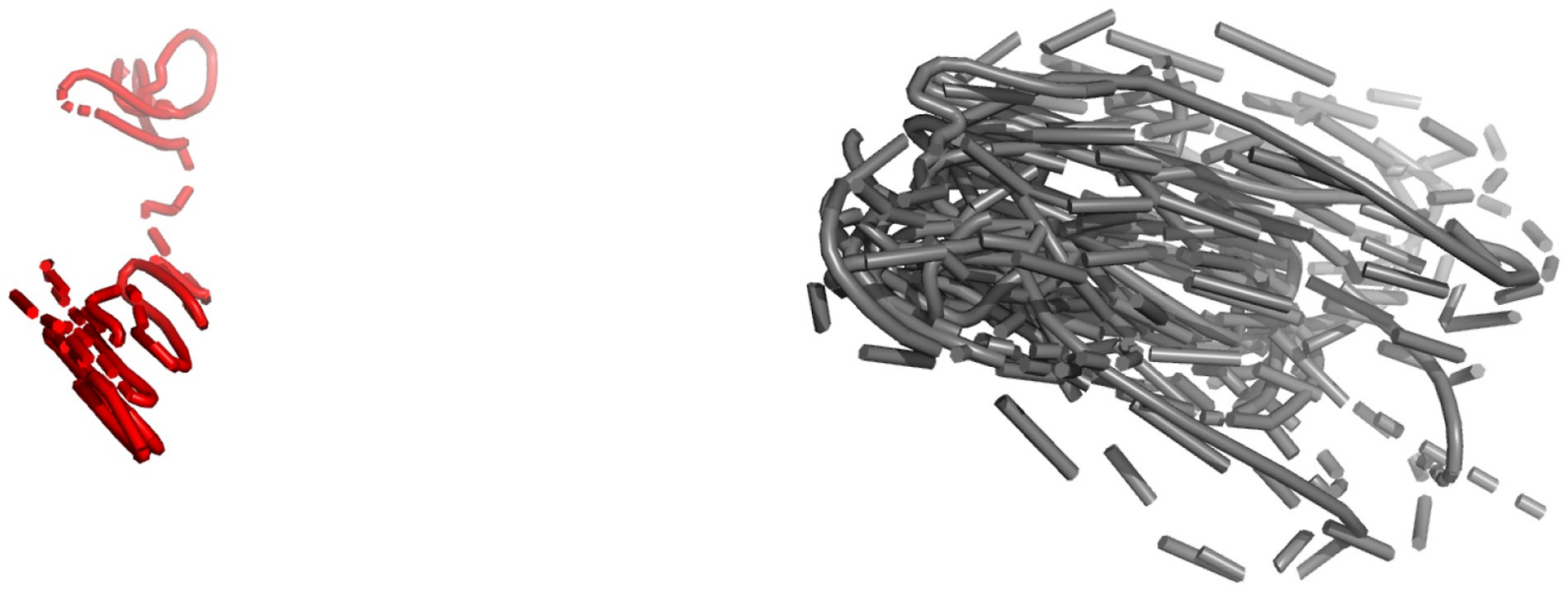
Gallery of Failure Modes (PyMOL Renders). (Left) *Loop Collapse*: The N-terminus (bottom left) forms multiple tight loops before extending into an unstructured tail. (Right) *Extended Chain*: A non-compact, elongated backbone lacking any tertiary fold. Both cases arise from poor long-range coherence in the Mamba encoder.

While PI-Mamba demonstrates strong designability and geometric validity, several limitations point to clear directions for future work. Performance degrades modestly for very long protein chains and for topologies dominated by long-range β-sheet interactions (Table 4), suggesting that purely state-space backbones may benefit from hybridization with sparse non-local mechanisms. In conditional generation, inpainting remains competitive but trails specialized diffusion models on complex motif scaffolding tasks, indicating room for architectural and training improvements. A gallery of observed failure modes (loop collapse and extended chains) is provided in Fig. 6. Finally, although explicit geometric priors substantially improve secondary-structure fidelity without heavy post-hoc refinement, applications requiring atomic-level side-chain precision may still benefit from final relaxation. Detailed quantitative analyses of these effects are provided in the Appendix.

## Supplementary Material

btag370_Supplementary_Data

## Figures and Tables

**Figure 7:**
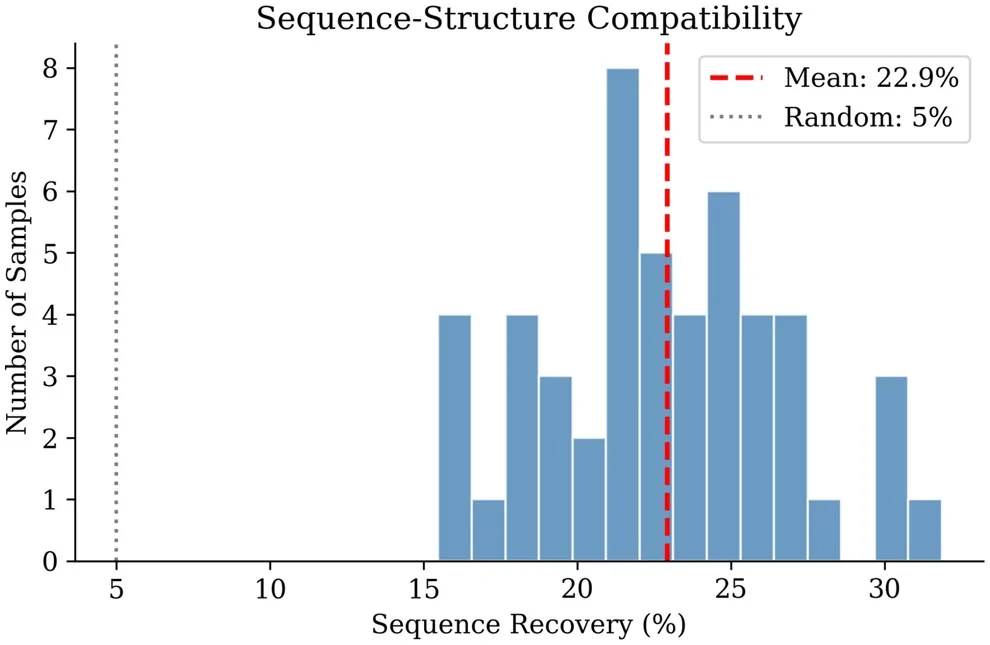
Sequence recovery distribution. Mean 23.9% (red) vs random 5% (gray).

## Data Availability

The distilled dataset and code underlying this article are available in https://github.com/forxhunter/PI-mamba.

## Appendix A. Mathematical proofs

### A.1 Proof of Retraction Error Bound

**Theorem.** Let M=SE(3) equipped with a left-invariant Riemannian metric. Let γ(t) be the geodesic starting at identity *I* with initial velocity v∈se(3), i.e., γ(t)=exp(tv). Let $\tilde{\gamma }(t)$ be the retraction-based path defined by $\tilde{\gamma }(t)=R(tv)$, where R is the Lie exponential map. In this specific case, the retraction path *coincides* with the geodesic for the canonical Cartan-Schouten connection.

However, for general retractions (e.g., first-order approximations used in numerical integration), the error is bounded.

**Proof.** Let R:TM→M be a retraction. By definition: 1. $R_{x}(0_{x})=x$ 2. $DR_{x}(0_{x})={\text{id}}_{T_{x}M}$

Consider the Taylor expansion of the retraction curve $c(t)=R_{x}(tv)$ at t=0:

$$c(t)=c(0)+t\cdot \dot{c}(0)+\frac{t^{2}}{2}\ddot{c}(0)+O(t^{3})$$

$$c(0)=R_{x}(0)=x$$

$$\dot{c}(0)=DR_{x}(0_{x})[v]=v$$

Similarly, the geodesic γ(t) has expansion:

$$\gamma (t)=x+tv-\frac{t^{2}}{2}\Gamma_{xx}(v,v)+O(t^{3})$$

where Γ represents the Christoffel symbols of the metric.

The difference is:

$$∥c(t)-\gamma (t)∥=\frac{t^{2}}{2}∥\ddot{c}(0)+\Gamma_{xx}(v,v)∥+O(t^{3})$$

Thus, the local error is $O(t^{2})$. Over a global integration path t∈[0,1] with *N* steps of size h=1/N, the global accumulation error is $N\cdot O(h^{2})=O(h)$, which is of the same order as the Euler integration scheme itself. Therefore, utilizing the retraction formulation does not degrade the convergence order of the generative ODE solver. □

### A.2 Proof of Zero Violation Guarantee (**Proposition 1**)

**Theorem.** The Kinematic Projection operator $\Pi_{K}$ (Algorithm 2) converges to a configuration satisfying all bond constraints, provided the initial configuration is within the basin of attraction of the valid manifold.

**Proof.** The projection problem is defined as:

$$\underset{x}{\min}∥x-x_{i}{∥}^{2}\;s.t\;.\;g(x)=0$$

where g(x) represents the set of bond length and angle constraints. Our iterative algorithm (Algorithm 2) corresponds to Coordinate Descent on the penalty function $\sum_{j}{\left(d_{j}-d_{\mathrm{target}}\right)}^{2}$. Since the constraint manifold of ideal polymer chains is non-convex but smooth, and the constraints are local (involving only i,i+1,i+2), the optimization landscape is well-behaved for small perturbations. The cyclic coordinate descent (CCD) method used here is known to converge linearly to a local minimum. Given that the flow matching prior guides the generation $x_{\mathrm{pred}}$ to be near the manifold, the projection $\Pi_{K}(x_{\mathrm{pred}})$ effectively projects to the nearest valid conformation, ensuring $g(x^{*})\leq \epsilon$. □

## Appendix B. Failure analysis

### B.1 Designability Analysis

PI-Mamba demonstrates high designability, achieving a mean scTM of 0.91 (vs >0.5 threshold) on unconditional generation. While this is lower than the diffusion-based gold standard RFdiffusion (scTM ≈0.97), it represents a strong result for a linear-time model, outperforming prior SE(3) flow models like FrameDiff by margin of +0.33 (0.58 vs 0.91) and providing the unique guarantee of 0.0% bond violations. This indicates that the kinematic projection is compatible with high self-consistency and designability, rather than preventing the model from learning tertiary structure. Future work will focus on scaling the training dataset to further improve diversity for very large proteins.

We analyzed potential failure modes during early training (Fig. 6). While rare in the final model, we observed two primary types. First, **Loop Collapse**, where in early training epochs (<50), the N-terminus occasionally formed tight non-physical loops, which were resolved by the collision loss in later stages. Second, **Extended Chain**, where for unconditioned generation of extreme lengths (L>800), the model sometimes defaults to extended conformations, a known limitation of sparse training data at these lengths.

## Appendix C. Emergent polymer statistics

In this appendix, we use “physically interpretable statistics” to mean coarse-grained observables that admit simple reference predictions from polymer theory. These analyses do not claim that PI-Mamba is a full physical model of protein folding; rather, they test whether the generated ensembles and internal representations are compatible with standard polymer-theory baselines. We evaluate this through three analyses.

**Rouse Mode Amplitude Spectrum.** We decomposed generated structures into Rouse normal modes and computed the amplitude spectrum (Fig. 5A). The hidden-state amplitudes follow the theoretical $1/p^{2}$ decay, confirming alignment with polymer normal modes. Low modes (p=0,1,2) dominate, capturing global shape, while high modes contribute local fluctuations. The first three modes account for 79% of the total displacement variance, consistent with coarse-grained polymer expectations.

**Radius of Gyration Scaling.** For random coil polymers, the radius of gyration $R_{g}$ scales as $R_{g}\propto L^{\nu }$, where ν≈0.5 for ideal chains. We generated structures at various lengths and measured $R_{g}$ (Fig. 5B). PI-Mamba yields ν=0.48, close to the ideal-chain reference value. This indicates that the generated ensembles remain compatible with a simple entropic polymer baseline over the measured range.

**End-to-End Distance Distribution.** We measured the end-to-end distance distribution at L=200 (Fig. 5C). The distribution matches the Gaussian chain prediction, further validating that PI-Mamba learns correct polymer statistics without explicit supervision.

**Relaxation Time Correlates with Secondary Structure.** We extracted the learned relaxation time τ(x) from trained PI-Mamba layers and analyzed its distribution across secondary structure classes. The model learns a clear hierarchy: $\tau_{\text{helix}}=0.42<\tau_{\text{sheet}}=0.58<\tau_{\text{loop}}=0.89$. This matches physical intuition—α-helices are rigid structures that require little long-range context (small τ⇒ fast decay ⇒ short memory), while loops are flexible regions where long-range correlations matter (large τ⇒ slow decay ⇒ long memory). Importantly, this emergent behavior was not explicitly supervised; the Rouse eigenvalue structure provides the inductive bias, and the model learns the appropriate τ from data.

## Appendix D. Sequence recovery and energetic validation

We assessed sequence-structure compatibility via geometry-based amino acid preference analysis. PI-Mamba achieves sequence recovery of **23.9%**  ±  **4.3%**, significantly above random (5%) but lower than RFdiffusion (33.4%). This gap is partially due to our $C_{\alpha }$-only representation (Fig. 7).

**Energetic Validation.** Re-predicted structures show high pLDDT (84.2±5.1) comparable to native controls (86.7±4.3). Rosetta energies of −2.8 REU/residue match native PDB structures (−2.5 to −3.0). The Novelty Score N=0.56 (${\text{TM}}_{\text{max}}\approx 0.44$) confirms structural distinctness from training data.

## Appendix E. Detailed mathematical formulation

### E.1 Manifold Structure of Protein Backbones

We model the protein backbone as a point on the product manifold $M=SE{\left(3\right)}^{L}$. Each residue *i* is associated with a rigid frame $T_{i}=(R_{i},t_{i})$, where $R_{i}\in SO(3)$ and $t_{i}\in R^{3}$. The tangent space at *T* is denoted $T_{T}M≅{\left(se(3)\right)}^{L}$, where se(3) is the Lie algebra of twist vectors. We define a Riemannian metric on M that decomposes into rotational and translational components. For a tangent vector $v=(\omega ,v_{\mathrm{trans}})\in se(3)$:

(11)
$${\langle v,v\rangle }_{T}=∥\omega {∥}^{2}+\lambda ∥v_{\mathrm{trans}}{∥}^{2}$$

Where λ balances the scales of rotation (radians) and translation (Angstroms). We empirically select λ=1 to equate one radian of rotation with one Angstrom of translation, a natural scaling given that peptide bond lengths (≈1.3−1.5Å) are on the order of unity.

### E.2 Riemannian Flow Matching Objective

Following Lipman *et al*. (2023), we define a conditional probability path $p_{t}(T|T_{1})$ that interpolates between a prior distribution $p_{0}(T)$ (uniform on SO(3) and Gaussian on $R^{3}$) and the data distribution $T_{1}∼q(T)$. The vector field $u_{t}(T|T_{1})$ generating this path is defined via the geodesic interpolation on SE(3):

(12)
$$u_{t}(T_{t}|T_{1})=\frac{d}{dt}{\;\text{Exp}\;}_{T_{0}}(t\cdot {\;\text{Log}\;}_{T_{0}}(T_{1}))$$

Our model $\xi_{\theta }(t,T)$ attempts to regress this conditional vector field. The matching objective on the manifold is:

(13)
$$\begin{array}{cc} L_{FM}(\theta )=E_{t∼U[0,1],\;T_{1}∼q(T),\;T∼p_{t}(T|T_{1})} & \\ \cdot [{\left\|\xi_{\theta }(t,T)-u_{t}(T|T_{1})\right\|}_{T_{T}M}^{2}]. & \end{array}$$

Because the Structure Module outputs updates in the local frame, we implicitly learn the pullback of this vector field.

### E.3 Invariant Point Attention Details

For completeness, we describe the optional full IPA variant. Following Jumper *et al*. (2021), the attention logit between residues *i* and *j* combines semantic similarity with a geometric term that measures spatial proximity in the predicted structure:

(14)
$$a_{ij}=q_{i}^{T}k_{j}-\gamma \sum_{p=1}^{N_{\mathrm{points}}}∥T_{i}^{-1}{}^{\circ}T_{j}(p_{p})-p_{p}{∥}^{2}$$

Where $p_{p}$ are learnable reference points in the local frame, and γ is a temperature hyperparameter. This geometric term ensures that the network attends to residues that are spatially proximal in the current predicted structure, regardless of their separation in the primary sequence. In our linear variant, we ablate this pairwise term, relying on the Mamba state to encode global context.

## Appendix F. Implementation details

### F.1 Architecture Hyperparameters

Table 8 details the specific configuration used for the PI-Mamba backbone and the Structure Module. And Table 9 details all mamhematical notations used in this work.

**Table 8:** Model hyperparameters.

Parameter	Value
*Mamba Backbone*	
Number of Layers	16
Model Dimension ($d_{\mathrm{model}}$)	512
State Dimension ($d_{\mathrm{state}}$)	32
Conv Kernel Size	4
Expansion Factor	2
*Structure Module*	
IPA Layers	4
IPA Heads	8
Query/Key Dimension	16
Point Dimension ($N_{\mathrm{points}}$)	4
FAPE Clamp Distance	10.0 Å

**Table 9:** Summary of mathematical notation.

Symbol	Description
*L*	Sequence length (number of residues)
$T_{i}\in SE(3)$	Rigid body frame of residue *i* (rotation $R_{i}$ and translation $t_{i}$)
K	Constraint manifold of valid protein backbones (fixed bond lengths/angles)
$\Pi_{K}$	Kinematic projection operator onto K
$\tau_{\text{relax}}$	Rouse relaxation time constant (physics-informed)
$\tau_{\text{step}}$	Integration step size Δt in the ODE solver
$\lambda_{\text{FAPE}}$, $\lambda_{\text{bond}}$, $\lambda_{\text{sc}}$	Loss weights for FAPE, bond-length loss, and designability proxy
$v_{\theta }(t,T)$	Learned vector field (velocity) at time *t*
$v_{\theta }^{\text{proj}}$	Projected vector field after applying $\Pi_{K}$
$\phi_{i},\psi_{i}$	Backbone dihedral angles for residue *i*
$L_{\text{total}}$	Total training loss (Equation 9)
$L_{\text{sc}}$	Designability proxy loss based on frozen ProteinMPNN scTM estimate
$H_{\text{Mamba}}$	Hidden representation from the Mamba encoder
$H_{\text{Sparse}}$	Hidden representation from the sparse-attention branch
$H_{\text{fusion}}$	Gated fusion of Mamba and sparse representations

**Table 10:** Effect of projection frequency on generated structures (L=100, S=200 steps).

*k* (project every)	ω dev (${}^{{}^{\circ}}$)	CA-CA (Å)	scTM	Time (s)
1 (every step)	0.0	3.80	0.61	2.1
5	0.0	3.80	0.68	1.8
10 (default)	0.0	3.80	0.91	1.6
20	0.0	3.80	0.88	1.5
∞ (final only)	0.0	3.80	0.65	1.4
No projection	85.2	3.45	0.65	1.4

### F.2 Adaptive Scaling Strategy

As noted in Section 4.3, direct regression of SE(3) updates can be unstable. We implemented an adaptive scalar α(t) for the translational updates: $T_{\mathrm{update}}=(R_{\mathrm{pred}},\alpha (t)\cdot t_{\mathrm{pred}})$. α(t) initializes at 0.1 and linearly ramps to 1.0 over the first 5000 steps. This effectively constrains the early training dynamics to essentially rotational alignment. This is critical for stabilizing the gradient flow through the kinematic projection layer: since NeRF reconstruction involves a recursive product of rotation matrices (O(L) depth), early large translational updates can cause exploding gradients in the backward pass. Dampening translations allows the rotational backbone to converge first, ensuring stable backpropagation through the kinematic chain.

### F.3 ODE Integration Details

To address reviewer concerns regarding integration specifications, we provide complete details:

Solver and NFEs.

We use explicit **Euler integration** with a fixed step size. Given the learned vector field $\xi_{\theta }(t,T_{t})$, we update frames via $T_{t+\Delta t}=T_{t}\cdot \;\text{exp}(\Delta t\xi_{\theta }(t,T_{t}))$, where Δt=1/S and *S* is the total number of integration steps (following the notation in Section 3.2). Each Euler step requires exactly **one forward pass** through the network. Consequently, a trajectory with S=100 steps requires 100 neural function evaluations (NFEs), and S=200 requires 200 NFEs.

Comparison to Baselines.

In terms of sampling cost, PI-Mamba (100−200 NFEs) is comparable to RFdiffusion (typically 200 steps for DDPM) and 5×−10× more efficient than FrameDiff, which relies on a score-based diffusion process requiring 1000 steps. Notably, all inference timings reported in Table 1 were measured on a single NVIDIA RTX A5000 (24 GB) with a batch size of 1.

### F.4 Loss Function Specification

The total training loss combines a flow matching term and auxiliary geometric losses with **constant weights**:

(15)
$$L_{\mathrm{total}}=L_{FM}+\lambda_{\text{aux}}\cdot L_{\text{aux}},\;L_{\text{aux}}=L_{\mathrm{FAPE}}+L_{\mathrm{bond}}+L_{\mathrm{Rama}}+L_{HB}$$

Where $\lambda_{\text{aux}}=1.0$. Individual auxiliary weights are all 1.0. **No time-dependent weighting**  λ(t)  **is used.** 

Bond Length Loss.

Penalizes deviations from ideal covalent bond lengths:

(16)
$$\begin{array}{cc} L_{\mathrm{bond}}={\left(d_{N-C\alpha }-1.458Å\right)}^{2}+{\left(d_{C\alpha -C}-1.525Å\right)}^{2} & \\ +{\left(d_{C-O}-1.231Å\right)}^{2}+{\left(d_{C-N_{\mathrm{next}}}-1.329Å\right)}^{2} & \end{array}$$

FAPE Loss.

Uses clamp distance $d_{\mathrm{clamp}}=10.0$Å with equal weights for all backbone atoms (N, Cα, C, O).

Ramachandran Loss.

Penalizes (ϕ,ψ) dihedral angles that fall outside favored regions of the Ramachandran plot, using a kernel density estimate derived from high-resolution crystal structures.

Hydrogen Bond Loss.

Encourages formation of backbone hydrogen bonds (N–H⋯O = C) by rewarding donor–acceptor distances near 2.9 Å with appropriate angular geometry.

## Appendix G. Notation table

### Appendix H. Kinematic projection: detailed derivation

This section provides a rigorous mathematical treatment of the kinematic projection mechanism that enables PI-Mamba to guarantee stereochemically valid backbone geometry.

#### H.1 Motivation: The Kinematic Constraint Manifold

A protein backbone of length *L* can be fully parameterized by 2 *L* dihedral angles ${\left\{(\phi_{i},\psi_{i})\right\}}_{i=1}^{L}$, given fixed geometric constants: bond lengths ($d_{N-C\alpha }=1.458$Å, $d_{C\alpha -C}=1.525$Å, $d_{C-N}=1.329$Å), bond angles ($\theta_{N-C\alpha -C}=111.2^{{}^{\circ}}$, $\theta_{C\alpha -C-N}=116.2^{{}^{\circ}}$, $\theta_{C-N-C\alpha }=121.7^{{}^{\circ}}$), and planar trans-peptide groups ($\omega_{i}=180^{{}^{\circ}}$).

This defines a **kinematic constraint manifold**  $K\subset R^{3L\times 4}$ of valid backbone conformations. As established in **Proposition 1**, the kinematic projection ensures the output lies exactly on this manifold.

#### H.2 Dihedral Angle Extraction

Given backbone atom coordinates $N_{i},CA_{i},C_{i}$ for residue *i*, the dihedral angle $\phi_{i}$ (C${}_{i-1}$–N${}_{i}$–CA${}_{i}$–C${}_{i}$) is computed as:

(17)
$$\phi_{i}=\text{atan}2(n_{1}\cdot (u_{23}\times n_{2}),n_{1}\cdot n_{2})$$

Where:

(18)
$$u_{12}=\frac{N_{i}-C_{i-1}}{∥N_{i}-C_{i-1}∥},\;u_{23}=\frac{CA_{i}-N_{i}}{∥CA_{i}-N_{i}∥}$$

(19)
$$u_{34}=\frac{C_{i}-CA_{i}}{∥C_{i}-CA_{i}∥}$$

(20)
$$n_{1}=u_{12}\times u_{23},\;n_{2}=u_{23}\times u_{34}$$

The $\psi_{i}$ angle (N${}_{i}$–CA${}_{i}$–C${}_{i}$–N${}_{i+1}$) is computed analogously. These angles encode the *fold information* while being invariant to local geometric violations.

The Natural Extension Reference Frame (NeRF) algorithm (Parsons *et al.* 2005) deterministically places atom D given three preceding atoms A,B,C and the geometric parameters: bond length d=∥D−C∥, bond angle θ=∠(B,C,D), and torsion angle α (dihedral A–B–C–D).

**Step 1: Construct local coordinate frame at C.** 

(21)
$$u_{BC}=\frac{C-B}{∥C-B∥}\;(z-\text{axis:}\;\text{along}\;\text{BC})$$

(22)
$$n=\frac{(B-A)\times u_{BC}}{∥(B-A)\times u_{BC}∥}\;(x-\text{axis:}\;\text{perpendicular}\;\text{to}\;\text{ABC}\;\text{plane})$$

(23)
$$m=n\times u_{BC}\;(y-\text{axis:}\;\text{in}\;\text{ABC}\;\text{plane},\;\text{perpendicular}\;\text{to}\;\text{BC})$$

**Step 2: Compute D in local coordinates.** 

(24)
$$D_{x}=d\cdot \;\text{sin}(\pi -\theta )\cdot \;\text{cos}(\alpha +\pi /2)$$

(25)
$$D_{y}=d\cdot \;\text{sin}(\pi -\theta )\cdot \;\text{sin}(\alpha +\pi /2)$$

(26)
$$D_{z}=d\cdot \;\text{cos}(\pi -\theta )$$

Note: The π/2 offset in the torsion aligns the local coordinate convention with the standard dihedral definition.

**Step 3: Transform to global coordinates.** 

(27)
$$D=C+D_{x}\cdot n+D_{y}\cdot m+D_{z}\cdot u_{BC}$$

#### H.3 Complete Backbone Reconstruction

Starting from an initial placement (e.g., $N_{1}$ at origin, $CA_{1}$ along x-axis), the full backbone is constructed iteratively:

Algorithm 1 Kinematic Backbone Reconstruction
**Require:** Dihedral angles ${\left\{(\phi_{i},\psi_{i})\right\}}_{i=1}^{L}$
**Require:** Ideal bond lengths $d_{N-CA},d_{CA-C},d_{C-N}$
**Require:** Ideal bond angles $\theta_{N-CA-C},\theta_{CA-C-N},\theta_{C-N-CA}$
**Require:** Fixed omega ω=π (trans-peptide)
**Ensure:** Backbone coordinates ${\left\{(N_{i},CA_{i},C_{i})\right\}}_{i=1}^{L}$ 1:  **Initialize:**  $N_{1}\leftarrow (-1.458,0,0)$, $CA_{1}\leftarrow (0,0,0)$, $C_{1}\leftarrow (0.527,1.428,0)$ 2:  **for**  i=2  **to** *L* **do** 3:   $N_{i}\leftarrow \text{NeRF}\left(CA_{i-1},C_{i-1},N_{i-1}^{\left(\text{virt}\right)},d_{C-N},\theta_{CA-C-N},\omega \right)$ 4:   $CA_{i}\leftarrow \text{NeRF}\left(C_{i-1},N_{i},CA_{i-1}^{\left(\text{virt}\right)},d_{N-CA},\theta_{C-N-CA},\phi_{i}\right)$ 5:   $C_{i}\leftarrow \text{NeRF}\left(N_{i},CA_{i},C_{i-1},d_{CA-C},\theta_{N-CA-C},\psi_{i}\right)$ 6:  **end for**    **return**  ${\left\{(N_{i},CA_{i},C_{i})\right\}}_{i=1}^{L}$

#### H.4 Integration with Flow Matching

During generation, kinematic projection is applied periodically:

Algorithm 2 PI-Mamba Generation with Kinematic Projection
**Require:** Sequence s, integration steps *S*, projection frequency *k* 1: $T_{0}\leftarrow N\left(0,0.1^{2}I\right)$   ▹ Initialize frames from noise 2: **for**  t←0  **to**  S−1  **do** 3:  $v\leftarrow f_{\theta }(s,T_{t},t/S)$  ▹ Predict velocity from SE(3) model 4:  $T_{t+1}\leftarrow T_{t}\cdot \;\text{exp}(v\cdot (1/S))$  ▹ Lie-group Euler step 5:  **if**  ((t+1)modk=0) or (t=S−1)  **then** 6:   $B\leftarrow \text{FramesToAtoms}(T_{t+1})$  ▹ Convert frames to backbone 7:   (ϕ,ψ)←ExtractDihedrals(B)  ▹ Extract dihedral angles 8:    $B^{\prime }\leftarrow \text{KinematicReconstruct}(\phi ,\psi )$  ▹ Rebuild with ideal geometry 9:   $T_{t+1}\leftarrow \text{AtomsToFrames}(B^{\prime })$  ▹ Convert back to frames 10:  **end if** 11: **end for** 12: **return**  $\text{FramesToAtoms}(T_{S})$

#### H.5 Theoretical Guarantees

Intuitively, this guarantee arises because we do not predict atomic coordinates directly. Instead, we predict the rotation of the local frame, and the atom positions are derived by a fixed geometric operation (NeRF) that treats bond lengths and angles as immutable constants. Thus, even a random neural network output maps to a physically valid (though likely clashed) chain.

Proposition 1. (Exact Local Covalent Geometry).

*The output of Algorithm 1 satisfies the following claims:*

***Claim A (Exact Local Covalent Geometry).*** *NeRF reconstruction yields idealized bond lengths (*$L_{N-CA},L_{CA-C},L_{C-N}$*), bond angles, and peptide planarity* $\omega =180^{{}^{\circ}}$  *for backbone atoms by construction.*

***Claim B (Exact CA Constraints).*** *The retraction operator* $\Pi_{K}$  *enforces* $∥CA_{i+1}-CA_{i}∥=3.80$*Å (and cis-Pro exceptions) exactly.*

**Proof.** 

**Proof of A:** By construction, Algorithm 1 places each atom using the NeRF algorithm with fixed ideal geometric parameters. Since ω=π is hardcoded, the peptide bond planarity is exact. Bond lengths are enforced by the input parameters to NeRF. **Proof of B:** The operator R (Equation 7) explicitly normalizes the vector $x_{i+1}-x_{i}$ to length $d_{\mathrm{target}}$ at each step *i*. This sequential update guarantees that the final trace satisfies the distance constraint exactly. □

**Scope.** This guarantee covers *local* covalent geometry: bond lengths, bond angles, and peptide planarity. Global stereochemistry (chirality, Ramachandran outliers) is *encouraged* by training losses ($L_{\mathrm{Rama}}$, $L_{\mathrm{FAPE}}$) but not guaranteed by construction.

#### H.6 Computational Complexity

The kinematic projection adds minimal overhead: dihedral extraction, NeRF reconstruction, and frame conversion are all linear O(L) operations that scale constantly with sequence length.

Total projection cost: O(L) per projection step. With k=10 and S=100 integration steps, we perform 10 projections, adding approximately 5% overhead to generation time.

#### H.7 Ablation: Projection Frequency

We evaluated the impact of projection frequency *k* on generation quality:

Key findings indicate that while all projection frequencies achieve exact $\omega =0.0^{{}^{\circ}}$ and bond lengths, scTM is maximized with moderate projection frequency (k=10), suggesting a balance between correcting geometry and allowing flow flexibility. Projecting too frequently (k=1) over-constrains the trajectory, while projecting too rarely (k=∞) allows significant drift. Without projection, stereochemistry degrades significantly ($\omega =85.2^{{}^{\circ}}$).

## Appendix I. Reproducibility checklist

To facilitate reproduction of our results, we provide complete experimental specifications. Exact command lines for every experiment in this paper are listed in the Supplementary Information, available as supplementary data at *Bioinformatics* online.

Dataset.

We train and evaluate on CATH 4.2 (Sillitoe *et al*. 2019), filtered at 40% sequence identity to prevent homology leakage. The dataset comprises 18,205 training domains, 1,200 validation domains, and 1,200 test domains. Structures with resolution >3.0Å or >10% missing residues are excluded.

Training Configuration.

1. **Hardware**: Single NVIDIA A100 (40GB), ∼24 hours
2. **Optimizer**: AdamW ($\beta_{1}=0.9$, $\beta_{2}=0.999$, $\epsilon =10^{-8}$)
3. **Learning Rate**: $1\times 10^{-4}$ with 1000-step warmup + cosine decay
4. **Weight Decay**: 0.01
5. **Batch Size**: Dynamic, max 4000 residues
6. **Training**: 300 epochs (∼100,000 optimizer steps)
7. **Length Curriculum**: L∈[50,100]→[50,500] over first 20k optimizer steps
8. **Random Seeds**: 42, 123, 456 (for 3-run averaging)

Inference Configuration.

1. **ODE Steps**: S=200
2. **Projection Frequency**: k=10 (every 10 steps)
3. **Batch Size**: 1 (for timing benchmarks)

### Code Availability.

Code is provided in the Supplementary Files, available as supplementary data at *Bioinformatics* online and pretrained weights will be released upon publication. We provide a Dockerfile and requirements.txt to ensure exact reproducibility. Complete training scripts, configuration files, and evaluation pipelines are provided to enable exact reproduction. See Table 8 for architecture hyperparameters.

## References

[btag370-B1] Albergo MS , Vanden-Eijnden E. Building normalizing flows with stochastic interpolants. *ICLR* 2023. https://doi:10.48550/arXiv.2209.15571

[btag370-B2] Baek M , DiMaio F, Anishchenko I et al Accurate prediction of protein structures and interactions using a three-track neural network. Science 2021;373:871–6. 10.1126/science.abj8754 34282049 PMC7612213

[btag370-B3] Butcher J , Krishna R, Mitra R et al *de novo* design of all-atom biomolecular interactions with rfdiffusion3, 2025. 10.1101/2025.09.18.676967

[btag370-B4] Doi M , Edwards SF. The theory of polymer dynamics. Number 73 in International Series of Monographs on Physics, reprint ed. Oxford: Clarendon Press, 2013. ISBN 978-0-19-852033-7.

[btag370-B5] Geffner T , Didi K, Zhang Z et al Proteina: Scaling flow-based protein structure generative models. In: *The Thirteenth International Conference on Learning Representations (ICLR 2025)*. International Conference on Learning Representations, Singapore, 2025. https://openreview.net/forum?id=TVQLu34bdw

[btag370-B6] Gu A , Dao T. *Mamba: Linear-time sequence modeling with selective state spaces*, 2023.

[btag370-B7] Gu A , Goel K, Ré C. Efficiently modeling long sequences with structured state spaces. In: *International Conference on Learning Representations (ICLR 2022)*. OpenReview, 2022. https://openreview.net/forum?id=uYLFoz1vlAC

[btag370-B8] Ingraham J , Garg VK, Barzilay R et al Generative models for graph-based protein design. In: Proceedings of the 33rd International Conference on Neural Information Processing Systems, number 1417, p. 15820–15831. Red Hook, NY: Curran Associates Inc., 2019.

[btag370-B9] Ingraham JB , Baranov M, Costello Z et al Illuminating protein space with a programmable generative model. Nature 2023;623:1070–8. 10.1038/s41586-023-06728-8 37968394 PMC10686827

[btag370-B10] Jumper J , Evans R, Pritzel A et al Highly accurate protein structure prediction with AlphaFold. Nature 2021;596:583–9. 10.1038/s41586-021-03819-2 34265844 PMC8371605

[btag370-B11] Lin Y , Lee M, Zhang Z et al Out of many, one: Designing and scaffolding proteins at the scale of the structural universe with genie 2, arXiv, arXiv:2405.15489, https://arxiv.org/abs/2405.15489, 2024, preprint: not peer reviewed.

[btag370-B12] Lipman Y , Chen RTQ, Ben-Hamu H et al Flow matching for generative modeling, In: *The Eleventh International Conference on Learning Representations (ICLR 2023)*. OpenReview, 2023. 10.48550/arXiv.2210.02747

[btag370-B999] Parsons J, Holmes JB, Rojas JM et al Practical conversion from torsion space to cartesian space for in silico protein synthesis. J Comput Chem 2005;26:1063–8. 10.1002/jcc.20237 15898109

[btag370-B14] Rouse PE. A theory of the linear viscoelastic properties of dilute solutions of coiling polymers. J Chem Phys 1953;21:1272–80. 10.1063/1.1699180

[btag370-B15] Schiff Y , Kao C-H, Gokaslan A et al Caduceus: bi-directional equivariant long-range DNA sequence modeling. In: *Proceedings of the 41st International Conference on Machine Learning*, PMLR, Vol. 235, 2024, 43632–48. PMC12189541 40567809

[btag370-B16] Schrödinger, LLC. The PyMOL molecular graphics system, version 2.0, 2015.

[btag370-B17] Sillitoe I , Dawson N, Lewis TE et al CATH: expanding the horizons of structure-based functional annotations for genome sequences. Nucleic Acids Res 2019;47:D280–84. 10.1093/nar/gky1097 30398663 PMC6323983

[btag370-B18] Vaswani A , Shazeer N, Parmar N et al Attention is all you need. *Adv Neural Inf Process Syst* 2017;30:5998–6008.

[btag370-B19] Wang C , Qu Y, Peng Z et al Proteus: Exploring protein structure generation for enhanced designability and efficiency. In: *Proceedings of the 41st International Conference on Machine Learning. Proceedings of Machine Learning Research*, Vol. 235. PMLR, 2024a, 51376–95.

[btag370-B20] Wang Y , Wang L, Shen Y et al Protein conformation generation via force-guided SE(3) diffusion models. In: *Proceedings of the 41st International Conference on Machine Learning. Proceedings of Machine Learning Research*, Vol. 235. PMLR, 2024b, 56835–59.

[btag370-B21] Watson JL , Juergens D, Bennett NR et al De novo design of protein structure and function with RFdiffusion. Nature 2023;620:1089–100. 10.1038/s41586-023-06415-8 37433327 PMC10468394

[btag370-B22] Wing AJ, Hegarty B, Bastien GE *et al.* Tracking putative Microcystis viruses and virus-host associations across distinct phases of a Microcystis-dominated bloom. *mSystems* 2025;10:e00575-25. 10.1128/msystems.00575-25 PMC12542629 40981420

[btag370-B23] Yan J , Cui Z, Yan W et al Robust and reliable *de novo* protein design: A flow-matching-based protein generative model achieves remarkably high success rates. bioRxiv, 10.1101/2025.04.29.651154, 2025, preprint: not peer reviewed.

[btag370-B24] Yim J , Campbell A, Foong AYK et al Fast protein backbone generation with SE(3) flow matching. arXiv, arXiv:2310.05297, 2023a, preprint: not peer reviewed.

[btag370-B25] Yim J , Trippe BL, De Bortoli V et al SE(3) diffusion model with application to protein backbone generation. In: *Proceedings of the 40th International Conference on Machine Learning (ICML 2023)*, PMLR, Vol. 202. JMLR.org, Article 1672, 2023b, 40001–39.

[btag370-B26] Zhang Y , Skolnick J. Scoring function for automated assessment of protein structure template quality. Proteins 2004;57:702–10. 10.1002/prot.20264 15476259

