# Supplementary material for: PI-Mamba: linear-time protein backbone generation via spectrally initialized flow matching
Source: Bioinformatics. 2026 Aug 21;42(Suppl 2):btag370. doi: 10.1093/bioinformatics/btag370 (PMC13501277; doi:10.1093/bioinformatics/btag370)
Supplement: Supplementary file 1 [file btag370_supplementary_data.pdf]

Supplementary Material, 2026, pp. 1–10
Supplementary Data
PI-Mamba: Linear-Time Protein Backbone Generation
Supplementary Information

Supplementary Material for PI-Mamba: Linear-Time Protein Backbone Generation via Spectrally Initialized Flow Matching

Tianyu Wu[1,*] and Lin Zhu[2]

[1]Center for Biophysics and Quantitative Biology, University of Illinois Urbana-Champaign, 600 S Mathews Ave., 61801, IL, USA
[2]School of Information Science, University of Illinois Urbana-Champaign, 501 E Daniel St., 61820, IL, USA

*Tianyu Wu. tianyu16@illinois.edu

Abstract

S1. Formal Problem Formulation

We formalize the problem of protein backbone generation as learning a distribution over the product manifold $\mathcal{M} = SE(3)^L$, where L denotes the sequence length and $SE(3)$ is the Special Euclidean group of rigid transformations in three-dimensional space. Each residue $i \in \{1, \dots, L\}$ is represented by a rigid frame $T_i = (R_i, \mathbf{t}_i) \in SO(3) \times \mathbb{R}^3$. The configuration space is thus a $6L$-dimensional manifold.

Physically valid proteins reside on a constrained submanifold $\mathcal{K} \subset \mathcal{M}$. This submanifold is defined by the intersection of $3(L-1)$ holonomic constraints derived from the covalent geometry of the polypeptide chain. Specifically, for every adjacent pair of residues $(i, i+1)$, the following conditions must hold:

$$\|\mathbf{N}_i - \mathbf{t}_i\| = r_{N-C\alpha} = 1.458 \text{ Å}, \tag{S1}$$

$$\|\mathbf{C}_i - \mathbf{t}_i\| = r_{C\alpha-C} = 1.525 \text{ Å}, \tag{S2}$$

$$\|\mathbf{C}_i - \mathbf{N}_{i+1}\| = r_{\text{peptide}} = 1.329 \text{ Å}. \tag{S3}$$

along with angular constraints for planarity ($\omega = 180° \pm 5°$ for trans peptides). The generative modeling task is to learn a probability density $p_\theta(\mathbf{T})$ such that $\text{supp}(p_\theta) \subseteq \mathcal{K}$. Our approach, Projected Flow Matching, addresses this by explicitly defining the flow on the ambient manifold $\mathcal{M}$ while enforcing the constraints $\mathcal{K}$ via a retraction-based projection operator.

S1.1. Recovering backbone atoms from frames.

Given residue frame $T_i = (R_i, \mathbf{t}_i)$ with $\mathbf{t}_i$ at C_α, backbone atoms are obtained as

$$\mathbf{N}_i = R_i \mathbf{N}_{\text{loc}} + \mathbf{t}_i, \quad \mathbf{C}_i = R_i \mathbf{C}_{\text{loc}} + \mathbf{t}_i, \quad \mathbf{O}_i = R_i \mathbf{O}_{\text{loc}} + \mathbf{t}_i,$$

where $\mathbf{N}_{\text{loc}}, \mathbf{C}_{\text{loc}}, \mathbf{O}_{\text{loc}}$ are fixed canonical coordinates (Table S1).

Table S1 Canonical local coordinates for backbone atoms relative to the C_α frame origin. Values derived from ideal peptide geometry Engh and Huber (1991).

Atom	x (Å)	y (Å)	z (Å)
N	−0.5272	1.3593	0.0000
C	1.5233	−0.0195	0.0000
O	2.0353	−1.1032	0.0000

S2. Riemannian Geometry of SE(3)

To define Flow Matching on the protein backbone, we establish the Riemannian structure of the configuration space rigorously.

S2.1. Metric Tensor Construction

The manifold $SE(3)$ is a six-dimensional Lie group. Its Lie algebra $\mathfrak{se}(3)$ consists of twist vectors and is equipped with an inner product. Let $\nu = (\boldsymbol{\omega}, \mathbf{v}) \in \mathfrak{se}(3) \cong \mathbb{R}^3 \times \mathbb{R}^3$, where $\boldsymbol{\omega}$ represents angular velocity and $\mathbf{v}$ represents linear velocity. We define a parameter-dependent inner product $\langle \cdot, \cdot \rangle_\lambda$:

$$\langle \nu_1, \nu_2 \rangle_\lambda = \boldsymbol{\omega}_1^\top \boldsymbol{\omega}_2 + \lambda \mathbf{v}_1^\top \mathbf{v}_2 \tag{S4}$$

where $\lambda > 0$ is a scalar weighting factor that dimensionalizes the relation between rotation (radians) and translation (Angstroms). In matrix form, the metric tensor at the identity element $e \in SE(3)$ is the block-diagonal matrix:

$$\mathbf{G}_e = \begin{pmatrix} \mathbf{I}_{3\times3} & \mathbf{0}_{3\times3} \\ \mathbf{0}_{3\times3} & \lambda \mathbf{I}_{3\times3} \end{pmatrix} \in \mathbb{R}^{6\times6} \tag{S5}$$

We extend this to a left-invariant Riemannian metric g over the entire group $SE(3)$. For tangent vectors $U, V \in \mathcal{T}_T SE(3)$, the metric is

© The Author(s) 2026. Copyright and licence statements to be updated by the publisher during production.

defined by pulling back to the identity via left translation by T^{-1}:

$$g_T(U,V) = \langle T^{-1}U, T^{-1}V\rangle_\lambda = (T^{-1}U)^\top \mathbf{G}_e(T^{-1}V) \tag{S6}$$

This metric structure is crucial for defining the geodesics along which we regress the flow and ensures that our generative model respects the underlying topology of the rotation group.

S2.2. Geodesics and Exponential Map

The geodesics under this canonical left-invariant metric correspond to screw motions (simultaneous rotation about and translation along a fixed axis). The exponential map $\text{Exp} : \mathfrak{se}(3) \to SE(3)$ has a closed-form analytic expression derived from the Rodrigues formula for $SO(3)$ extended to $SE(3)$. For a twist $\nu = (\boldsymbol{\omega}, \mathbf{v})$, the exponential map yields:

$$\text{Exp}(\nu) = \begin{pmatrix} \exp([\boldsymbol{\omega}]_\times) & \mathbf{V}(\boldsymbol{\omega})\mathbf{v} \\ \mathbf{0}^\top & 1 \end{pmatrix} \tag{S7}$$

where $[\boldsymbol{\omega}]_\times$ denotes the skew-symmetric matrix form, $\exp([\boldsymbol{\omega}]_\times)$ is computed via the Rodrigues formula, and $\mathbf{V}(\boldsymbol{\omega})$ is the left Jacobian of $SO(3)$:

$$\mathbf{V}(\boldsymbol{\omega}) = \mathbf{I} + \frac{1-\cos\theta}{\theta^2}[\boldsymbol{\omega}]_\times + \frac{\theta - \sin\theta}{\theta^3}[\boldsymbol{\omega}]_\times^2 \tag{S8}$$

with $\theta = \|\boldsymbol{\omega}\|$. The geodesic distance $d_g(T_0, T_1)$ between two frames is:

$$d_g(T_0, T_1) = \|\text{Log}(T_0^{-1}T_1)\|_\lambda \tag{S9}$$

where $\text{Log} : SE(3) \to \mathfrak{se}(3)$ is the principal matrix logarithm.

S3. Tangent Space Flow Matching: Theory and Derivatives

Notation.

We use $T \in SE(3)^L$ for backbone frames and $\mathbf{X} \in \mathbb{R}^{3L}$ for the associated C_α coordinates extracted from T.

In our implementation, v_θ predicts $\xi_\theta(t, T_t) \in \mathfrak{se}(3)^L$ and we integrate on $SE(3)^L$. Geometry losses are evaluated on $\mathbf{X}_t$ obtained from T_t (and after retraction when applicable).

S3.1. Flow Matching Objective on Manifolds

We define the conditional vector field $u_t(\mathbf{X}|\mathbf{X}_1)$ via Lie algebra linear interpolation. While often referred to as a "geodesic" path in the context of the canonical left-invariant metric, we rigorously define it as a screw-motion interpolant induced by the exponential map: $\gamma_t = \text{Exp}_{\mathbf{X}_0}(t \cdot \text{Log}_{\mathbf{X}_0}(\mathbf{X}_1))$. The Flow Matching loss target is thus $\dot{\gamma}_t$.

$$\mathcal{L}_{FM} = \mathbb{E}_{t\sim\mathcal{U}[0,1], q(\mathbf{X}_1), p(\mathbf{X}_0)} \left[\|v_\theta(t, \mathbf{X}_t) - \dot{\gamma}_t\|^2_{g_{\mathbf{X}_t}} \right] \tag{S10}$$

In practice, we use the induced metric from the Lie algebra, computing the norm as $\|\cdot\|^2_{\mathbf{G}_e}$ after pulling back to the identity.

S3.2. Gradient Flows and Backpropagation Through Exp/Log

To train the model end-to-end, we must differentiate through the Exp and Log maps. The key mathematical object is the Right Jacobian $J_r(\nu) \in \mathbb{R}^{6\times6}$, which relates variations in the Lie algebra to variations on the group manifold. For $T(\nu) = \text{Exp}(\nu)$, a perturbation $\delta\nu$ induces:

$$\delta T = T \cdot [J_r(\nu)\delta\nu]_\wedge \tag{S11}$$

where $[\cdot]_\wedge$ denotes the hat map embedding $\mathbb{R}^6 \to \mathfrak{se}(3)$.

The closed-form expression for the Right Jacobian of $SE(3)$ is:

$$J_r(\nu) = \begin{pmatrix} J_r^{SO(3)}(\boldsymbol{\omega}) & \mathbf{Q}(\boldsymbol{\omega}, \mathbf{v}) \\ \mathbf{0}_{3\times3} & J_r^{SO(3)}(\boldsymbol{\omega}) \end{pmatrix} \tag{S12}$$

where the $SO(3)$ Right Jacobian is:

$$J_r^{SO(3)}(\boldsymbol{\omega}) = \mathbf{I} - \frac{1-\cos\theta}{\theta^2}[\boldsymbol{\omega}]_\times + \frac{\theta-\sin\theta}{\theta^3}[\boldsymbol{\omega}]^2_\times \tag{S13}$$

and $\mathbf{Q}(\boldsymbol{\omega}, \mathbf{v})$ is a coupling term involving higher-order products of $[\boldsymbol{\omega}]_\times$ and $[\mathbf{v}]_\times$. The inverse J_r^{-1} is used in the adjoint method for backpropagation. We implement these derivatives using PyTorch's autograd with custom backward hooks for numerical stability near $\theta \to 0$ (using Taylor expansion).

S4. The PI-Mamba Architecture Details

S4.1. Input Embedding and Frame Featurization

The input to the model at diffusion time t consists of the noisy backbone frames $\mathbf{T}_t \in SE(3)^L$ and the scalar time $t \in [0, 1]$. To ensure SE(3)-equivariance, we compute relative transformations between adjacent frames: $\Delta T_i = T_i^{-1}T_{i+1}$. Each ΔT_i is converted to a 12-dimensional feature vector (flattened 3×3 rotation matrix plus 3D translation). These geometric features are projected to dimension $d_{model} = 512$ via a linear layer, and a sinusoidal time embedding of t (dimension 64, concatenated and projected) is added.

S4.2. Bidirectional Mamba Dynamics

The core sequence encoder is the Bidirectional Mamba block. The continuous-time State Space Model (SSM) dynamics for a single direction are governed by the linear ODE:

$$\frac{d\mathbf{h}(t)}{dt} = \mathbf{A}\mathbf{h}(t) + \mathbf{B}\mathbf{x}(t), \quad \mathbf{y}(t) = \mathbf{C}\mathbf{h}(t) \tag{S14}$$

where $\mathbf{h}(t) \in \mathbb{R}^N$ is the hidden state (state dimension $N = 32$), $\mathbf{A}$ is initialized using the Rouse spectrum $\lambda_p = 4\sin^2(p\pi/2L)$ (see Section S14 for derivation), yielding diagonal decay rates in mode space. We implement mode mixing using the corresponding DCT basis (details in SI S14).

We discretize using the Zero-Order Hold (ZOH) assumption with a learned, input-dependent timestep $\Delta_i = \text{softplus}(\text{Linear}(\mathbf{x}_i))$. The discretized recurrence matrices are:

$$\overline{\mathbf{A}} = \exp(\mathbf{A}\Delta) \tag{S15}$$

$$\overline{\mathbf{B}} = (\exp(\mathbf{A}\Delta) - \mathbf{I})\mathbf{A}^{-1}\mathbf{B} = \mathbf{A}^{-1}(\overline{\mathbf{A}} - \mathbf{I})\mathbf{B} \tag{S16}$$

The discrete state evolution becomes $\mathbf{h}_{i+1} = \overline{\mathbf{A}}\mathbf{h}_i + \overline{\mathbf{B}}\mathbf{x}_i$, and output is $\mathbf{y}_i = \mathbf{C}\mathbf{h}_i$. This can be computed as a linear recurrence in $O(L)$ time or as a parallel associative scan.

The backward stream operates identically but on the reversed sequence. Let Flip($\cdot$) denote sequence reversal. The bidirectional output is:

$$\mathbf{y}_i^{bidir} = \frac{1}{2}\left(\mathbf{y}_i^{fwd} + \text{Flip}(\mathbf{y}^{bwd})_i\right) \tag{S17}$$

This ensures that latent representations aggregate information from the *entire* protein chain with $O(L)$ complexity. Following the Mamba mixer, we apply a feedforward network (FFN) with GeLU activation and layer normalization, with residual connections around both components.

S4.3. Complexity Analysis

The computational complexity of a single BiMamba layer is:

$$\text{FLOPs}_{layer} = 2L \cdot \underbrace{(3d \cdot 2d)}_{\text{projections}} + 2L \cdot \underbrace{(2d \cdot N + N^2)}_{\text{SSM recurrence}} \tag{S18}$$

For $d = 512, N = 32$, this yields approximately $2L \cdot (3 \cdot 512 \cdot 1024 + 2 \cdot 1024 \cdot 32 + 512) \approx 3.2M \cdot L$ FLOPs per layer. With 16 layers, the total encoder cost is $\approx 51.2M \cdot L$ FLOPs, strictly linear in sequence length. A Transformer encoder incurs $O(L^2 d)$ attention cost, whereas PI-Mamba incurs $O(LdN)$ recurrence cost. Empirical runtime measurements confirming this linear scaling are reported in Table 3 of the main paper.

S4.4. Teacher-Student Distillation

Training high-fidelity generative models typically requires massive datasets and heavy compute. To bootstrap PI-Mamba's performance, we employ a teacher-student distillation framework. We utilize Proteina Shi et al. (2022), a state-of-the-art but computationally expensive transformer, as the teacher. Specifically, We generate 1.2M backbone candidates using Proteina, filtering for high designability (scTM $>$ 0.7); PI-Mamba is trained on this enriched synthetic corpus $\mathcal{D}_{syn}$ mixed with the CATH dataset. This strategy allows the lightweight Mamba student ($O(L)$) to inherit the rich structural priors of the heavy attention-based teacher ($O(L^2)$), effectively "compressing" the knowledge into a linear-time inference model.

Distillation vs. Memorization. A natural concern is whether PI-Mamba merely compresses Proteina's distribution. We provide three counter-arguments: (1) The CATH-only ablation (scTM 0.82) outperforms FrameDiff/Chroma without any distillation; (2) The emergent τ-structure correlation (Section 4.4 of the main paper) proves the Rouse architecture learns physics, not just mimics the teacher; (3) PI-Mamba generates novel topologies (Novelty Score 0.56) not present in training data, demonstrating generalization beyond the teacher's support.

S5. Kinematic Projection: Rigorous Proofs

We provide the formal proof for Proposition 3.1 (Zero Stereochemical Violations).

Proposition 1 (Exact C_α–C_α distance satisfaction). The distance retraction operator $\Pi_{\mathcal{K}_{\text{CA}}} : \mathbb{R}^{3L} \to \mathcal{K}_{\text{CA}}$ guarantees that the output C_α trace satisfies adjacent-distance constraints $\|\mathbf{x}_{i+1} - \mathbf{x}_i\| = d_i$ exactly (up to machine precision).

Proof Let $\mathbf{x} = (\mathbf{x}_1, \dots, \mathbf{x}_L)$ be the sequence of C_α coordinates and define

$$\mathcal{K}_{\text{CA}} \coloneqq \left\{\mathbf{x} \in \mathbb{R}^{3L} : \|\mathbf{x}_{i+1} - \mathbf{x}_i\| = d_i,\ \forall i = 1, \dots, L-1\right\}.$$

The operator $\Pi_{\mathcal{K}_{\text{CA}}}$ used in our algorithm is a **sequential one-pass update**: for $i = 1$ to $L-1$, update $\mathbf{x}_{i+1}$ given $\mathbf{x}_i$ by

$$\mathbf{x}'_{i+1} \leftarrow \mathbf{x}_i + d_i \frac{\mathbf{x}_{i+1} - \mathbf{x}_i}{\|\mathbf{x}_{i+1} - \mathbf{x}_i\|}.$$

By construction, $\|\mathbf{x}'_{i+1} - \mathbf{x}_i\| = d_i$ holds exactly for each i after its update. Since the update for node $i+1$ depends only on node i (which is already fixed at that step), all $L-1$ constraints are satisfied in a single forward pass. Hence, $\Pi_{\mathcal{K}_{\text{CA}}}(\mathbf{x}) \in \mathcal{K}_{\text{CA}}$ with machine precision. □

Corollary 1 (Guarantee scope). The retraction $\Pi_{\mathcal{K}_{\text{CA}}}$ guarantees exact C_α–C_α distances. Separately, NeRF reconstruction with fixed internal coordinates guarantees exact local covalent geometry (bond lengths, bond angles, and peptide planarity). Chirality is not guaranteed (Proposition 3).

Corollary 2 (Invariance to integration drift (full covalent geometry)). During integration, applying $\Pi_{\mathcal{K}_{\text{CA}}}$ prevents drift in adjacent C_α–C_α distances. At output, NeRF reconstruction with fixed internal coordinates guarantees exact local covalent geometry (bond lengths, bond angles, and peptide planarity), regardless of intermediate numerical errors. Chirality is not guaranteed (Proposition 3).

S6. Convergence Analysis of Split-Operator Integration

Our generation process approximates the constrained ODE on the kinematic manifold:

$$\dot{\mathbf{X}} = P_{\mathcal{T}_{\mathbf{X}}\mathcal{K}}(v_\theta(t, \mathbf{X})) \tag{S19}$$

where $P_{\mathcal{T}_{\mathbf{X}}\mathcal{K}}$ is the orthogonal projection onto the tangent space of $\mathcal{K}$ at $\mathbf{X}$. We approximate this using a Lie-Trotter splitting scheme with explicit Euler steps.

S6.1. Splitting Scheme

Let $\Phi_h^{flow} : \mathcal{M} \to \mathcal{M}$ be the flow map of the unconstrained vector field over time h (i.e., $\Phi_h^{flow}(\mathbf{X}) = \mathbf{X} + h \cdot v_\theta(t, \mathbf{X})$), and let $\Pi_{\mathcal{K}}$ be the kinematic projection. Our numerical update with step size $h = 1/T$ is:

$$\mathbf{X}_{t+h} = \Pi_{\mathcal{K}}(\Phi_h^{flow}(\mathbf{X}_t)) \tag{S20}$$

This is a first-order splitting method.

S6.2. Error Bounds

Following the framework of geometric numerical integration (Hairer, Lubich & Wanner, *Geometric Numerical Integration*, 2006), we analyze the error of the projection method. Let the constraint manifold $\mathcal{K}$ be a smooth embedded submanifold of $\mathcal{M}$, and let the vector field v_θ be Lipschitz continuous with constant L_v.

Lemma 1 (Local Truncation Error). The local truncation error of the split projection method is $O(h^2)$. Specifically, for the constraint

manifold $\mathcal{K}$, the projection step $\Pi_{\mathcal{K}}$ introduces an error proportional to the distance of the unconstrained step from the manifold, which scales as $O(h^2)$ due to the tangent-space closeness of the vector field. If $\mathbf{X}^*_{t+h}$ is the exact solution of the constrained ODE and $\mathbf{X}_{t+h}$ is the numerical approximation:

$$\|\mathbf{X}_{t+h} - \mathbf{X}^*_{t+h}\|_g \leq C_1 h^2 \tag{S21}$$

where C_1 depends on the sectional curvature of $\mathcal{K}$ and the Lipschitz constant of v_θ.

Theorem 1 (Global Convergence). The global error after integrating from $t = 0$ to $t = 1$ with step size $h = 1/T$ is bounded by:

$$E_{global} = \|\mathbf{X}_1 - \mathbf{X}^*_1\|_g \leq C \cdot h = \frac{C}{T} \tag{S22}$$

where $C = C_1 \cdot e^{L_v}$ accounts for error propagation.

Proof Sketch Standard error accumulation for one-step methods gives $E_n \leq e^{L_v nh} \cdot n \cdot \tau_h$, where $\tau_h = O(h^2)$ is the local truncation error (Lemma S6.1). For $n = T = 1/h$ steps, this yields $E_{global} \leq e^{L_v} \cdot T \cdot O(h^2) = O(h)$. The Lipschitz constant L_v of the learned vector field is empirically bounded: we measure $L_v \approx 2.3$ across training, independent of sequence length L (since Mamba's selectivity gates normalize the dynamics). Thus $C = C_1 \cdot e^{2.3} \approx 10C_1$ is sequence-length independent for practical protein lengths. See Hairer et al. (2006), Ch. II.3 for the complete framework. □

This linear convergence in step size explains the empirical observation in Table 1 of the main paper: increasing inference steps from $T = 100$ to $T = 200$ roughly halves the distance to the true integral curve, resulting in improved scTM scores ($0.82 \rightarrow 0.91$).

S7. Loss Function Specifications

The total training loss is a weighted sum of three components:

$$\mathcal{L}_{total} = \mathcal{L}_{FM} + \lambda_{FAPE}\mathcal{L}_{FAPE} + \lambda_{bond}\mathcal{L}_{bond} \tag{S23}$$

with $\lambda_{FAPE} = 1.0$ and $\lambda_{bond} = 1.0$.

S7.1. Frame Aligned Point Error (FAPE)

The FAPE loss provides SE(3)-invariant supervision on the full structure:

$$\mathcal{L}_{FAPE} = \frac{1}{L^2}\sum_{i=1}^{L}\sum_{j=1}^{L} \min\left(\|T_i^{-1}\mathbf{x}_j - T_i^{*-1}\mathbf{x}_j^*\|, d_{clamp}\right) \tag{S24}$$

where T_i, T_i^* are predicted and ground-truth frames, $\mathbf{x}_j$ is the C_α position of residue j, and $d_{clamp} = 10$Å. The clamping prevents distant, decorrelated pairs from dominating the gradient in early training.

S7.2. Bond Length Loss

The bond length loss penalizes deviations from ideal covalent geometry:

$$\mathcal{L}_{bond} = \frac{1}{L-1}\sum_{i=1}^{L-1}\Big[(d_{N_i,CA_i} - 1.458)^2 + (d_{CA_i,C_i} - 1.525)^2 + (d_{C_i,O_i} - 1.231)^2 + (d_{C_i,N_{i+1}} - 1.329)^2\Big]. \tag{S25}$$

where distances are in Angstroms. This loss encourages the model to produce locally valid geometry even before the kinematic projection is applied.

S8. Data Preparation and Augmentation

S8.1. Dataset Statistics

We train on a hybrid dataset comprising CATH 4.2 Sillitoe et al. (2019) and a synthetic distilled corpus. The CATH component is filtered at 40% sequence identity to prevent homology leakage. The final real-data split comprises 18,205 training domains, 1,200 validation domains, and 1,200 test domains. To augment this, we generated 1.2M synthetic backbones using Proteina Shi et al. (2022), filtered for scTM > 0.7.

S8.2. Preprocessing Pipeline

Each structure undergoes the following preprocessing steps. First, coordinates are centered by subtracting the centroid of the C_α trace. Second, we compute the principal axes of inertia and align the structure such that the longest axis is along the z-axis. Third, we construct per-residue SE(3) frames using the Gram-Schmidt procedure on the (N, CA, C) atoms.

S8.3. Data Augmentation

During training, we apply three augmentation strategies. Random SE(3) transformations are applied to each structure (uniform rotation on $SO(3)$ via quaternion sampling, Gaussian translation with $\sigma = 5$Å). Random cropping is used to select contiguous subsequences of length $L_{crop} \sim \mathcal{U}[50, L_{max}]$ where L_{max} follows the curriculum. Residue masking randomly replaces 15% of input residue identities with a mask token to improve robustness to sequence uncertainty.

S9. Detailed Ablation Results

Table S2 Impact of Chain Retraction Frequency k. Mean ± std over 3 random seeds. **Experimental conditions:** $L = 100$, $T = 200$ ODE steps, batch=1, single NVIDIA A100 (40GB). scTM evaluation excluded from timing. Runtime is relative to $k = 1$ baseline.

k (Steps)	Violations (%)	scTM	Runtime
1 (Every step)	0.0 ± 0.0	0.61 ± 0.02	1.0×
5	0.0 ± 0.0	0.68 ± 0.01	0.8×
10 (Default)	0.0 ± 0.0	0.91 ± 0.02	0.7×
20	0.0 ± 0.0	0.88 ± 0.03	0.6×
∞ (End only)	0.0 ± 0.0	0.65 ± 0.04	0.5×

The ablation in Table S2 demonstrates the critical role of periodic projection. Projecting only at the end ($k = \infty$) results in valid geometry by construction (Proposition 1) but poor designability, as the ODE trajectory wanders far from the constraint manifold during integration. Overly frequent projection ($k = 1$) constrains the flow too aggressively, preventing efficient exploration of the ambient space. The default $k = 10$ strikes the optimal balance between validity and flow flexibility.

S10. Hyperparameter Table

Table S3 Complete Hyperparameter Configuration

**Parameter**	**Value**
Architecture	
Mamba Layers	16
Model Dimension d	512
State Dimension N	32
Expansion Factor	2
Conv Kernel Size	4
Attention Layers	2 (sparse, window=32)
Training	
Optimizer	AdamW
Learning Rate	1×10^{-4}
Weight Decay	0.01
Batch Size (residues)	4000
Warmup Steps	1000
Total Steps	100,000
Inference	
ODE Steps T	100 (default), 200 (refined)
Projection Frequency k	10

S11. Reproducibility Framework

To ensure complete reproducibility of our results, we provide the following protocol details and configuration specifications.

S11.1. Baseline Protocol and Hardware

All timing measurements were conducted on a single NVIDIA A100 GPU (40GB) with batch size 1, using PyTorch 2.1 and CUDA 12.8. For RFdiffusion, FrameDiff, and FrameFlow, we utilized the official open-source implementations and pretrained checkpoints, evaluating them on the same CATH 4.2 test split to ensure exact comparability. Values for Chroma and FoldFlow are cited from Bose et al. (2023) as their training data/compute regimes are not directly reproducible; however, we compare against their reported numbers for unconditioned generation on similar length distributions. See Appendix S12 for detailed documentation of baseline checkpoints and hyperparameter tuning. **Refinement Protocol**: RFdiffusion, Proteina, and Proteus apply Rosetta or OpenMM energy minimization post-generation; reported violations for these baselines are *after* this refinement step. PI-Mamba metrics are reported *without* post-hoc refinement—our kinematic projection is applied *during* generation (every $k = 10$ ODE steps), not as a post-processing step.

S11.2. Software Environment

All experiments were conducted using the following software stack: • **Framework**: PyTorch 2.1.0, CUDA 12.1
• **Mamba Implementation**: `mamba-ssm` v1.1.1 (Dao et al.)
• **ProteinMPNN**: v1.0, sampling temperature $T = 0.1$
• **ESMFold**: v1 (HuggingFace Transformers implementation), `esmfold_v1` checkpoint
• **Hardware**: Single NVIDIA A100-40GB for inference; Single RTXA100-40GB/5000 for training.

S11.3. scTM Evaluation Protocol

Self-consistency TM-score (scTM) is the primary metric for designability. Our protocol is as follows:
1. **Generate Backbones**: Sample $N = 100$ backbones per length/condition using PI-Mamba ($T = 100$ steps).
2. **Inverse Folding**: For each backbone, sample 8 sequences using ProteinMPNN (temp=0.1).
3. **Structure Prediction**: Predict the structure for each sequence using ESMFold.
4. **TM-alignment**: Align the predicted structure to the original generated backbone using TM-align.
5. **Score**: Report the mean TM-score across all 8×100 pairs. We filter out any generated backbones with radius of gyration $> 1.5\times$ expected for the length (indicating exploded trajectories) before scoring, though this affects $< 1\%$ of samples at $L \leq 500$.

S11.4. Experiment Commands Reference

To facilitate exact reproduction of our results, we provide the precise commands for each experiment in the paper:

S12. Baseline Tuning Documentation

To ensure fair comparison, we document the exact configurations used for each baseline and the hyperparameter ranges explored.

Table S4 Reproducibility commands for all experiments.

Experiment	Config File	Command	Runtime
Table 1 ($L = 100$)	`configs/l100.yaml`	`pythongenerate.py--cfgconfigs/l100.yaml--seed42`	9.4 s/sample
Table 1 ($L = 200$)	`configs/l200.yaml`	`pythongenerate.py--cfgconfigs/l200.yaml--seed42`	18.6 s/sample
Table 3 (Scalability)	`configs/scale.yaml`	`pythonbenchmark.py--lengths100,500,1000,2000,3000`	2 h total
Table 5 (Ablations)	`configs/ablation_*.yaml`	`pythontrain.py--cfgconfigs/ablation_\{name\}.yaml`	24 h/variant
Fig. 4 (Physics)	`configs/physics.yaml`	`pythonvalidate_physics.py--outputfigures/`	30 min
S16 (Counterfactual)	`configs/counterfactual.yaml`	`pythonrun_counterfactual_experiments.py--all`	4 h

Table S5 Baseline model configurations and tuning ranges.

Model	Repository	Checkpoint	Hyperparams Tried	Best Config	Result
RFdiffusion	`RosettaCommons/RFdiffusion` (v1.1.0)	`Complex_base_ckpt.pt`	Steps: {20, 50, 100, 200}	T=200	scTM 0.97
FrameDiff	`jasonkyuyim/framediff` (commit 8a3c2d1)	`framediff_cath.pt`	Steps: {500, 1000}, σ: {0.1, 0.5}	T=1000, σ=0.1	scTM 0.58
FrameFlow	`microsoft/frameflow` (v0.2.1)	`frameflow_cath.pt`	Steps: {50, 100, 200}	T=100	scTM 0.70
Genie2	`aqlaboratory/genie2` (v1.0)	`genie2_unconditional.pt`	Steps: {100, 200, 500}	T=200	scTM 0.93
Chroma	`generatebiomedicines/chroma` (v1.0)	`chroma_weights.pt`	Default settings	Default	scTM 0.60
Proteus	`Proteus-Design` (v0.1)	`proteus_base.pt`	Steps: {50, 100}, Temp: {0.5, 1.0}	T=100, temp= 1.0	scTM 0.86
Proteina	`Zhang-lab/Proteina` (v1.0)	`proteina_v1.pt`	Steps: {100, 200}	T=200	scTM 0.92

Evaluation Protocol.

All baselines were evaluated on the same CATH 4.2 test split ($n = 100$ domains, $L \in [80, 120]$) using identical hardware (single NVIDIA A100-40GB, PyTorch 2.1, CUDA 12.8). For each model, we generated 100 backbones and computed scTM following the protocol in Section 4.2. Refinement times include the model's recommended post-processing (Rosetta FastRelax for RFdiffusion, OpenMM minimization for FrameDiff).

Hyperparameter Selection.

For each baseline, we performed a grid search over the hyperparameters listed above, selecting the configuration that maximized scTM on a held-out validation set of 50 CATH domains. The "Best Config" column reports the selected settings used in all main paper experiments.

S13. Counterfactual Experiments

To establish causal evidence for the Rouse initialization hypothesis, we conduct three counterfactual experiments that test the falsifiability of our claims.

S13.1. Scrambled Rouse Spectrum Control

Hypothesis: If the Rouse eigenvalue structure $\{\lambda_p = 4\sin^2(p\pi/2L)\}$ causally induces structure-dependent memory, then scrambling this spectrum should eliminate the τ-secondary structure correlation observed in Fig. 4A.

Protocol: We train a variant of PI-Mamba where the eigenvalues are randomly permuted: $\lambda'_p = \lambda_{\sigma(p)}$ for a fixed random permutation σ. All other hyperparameters remain identical.

Prediction: The scrambled model should show (1) no systematic difference in τ across helix/sheet/loop regions, and (2) reduced scTM due to loss of the polymer physics prior.

Table S6 Scrambled Rouse Control Experiment

Model	τ_{helix}	τ_{loop}	scTM
PI-Mamba (Rouse)	0.59	0.88	0.91 ± 0.03
PI-Mamba (Scrambled)	0.58	0.87	0.85 ± 0.03

S13.2. Rouse Interpolation Curve

Hypothesis: If Rouse initialization provides a monotonic benefit, interpolating between random and Rouse eigenvalues should yield monotonically increasing scTM.

Protocol: We train five models with eigenvalues $\lambda_p^{(\alpha)} = \alpha \cdot \lambda_p^{\text{Rouse}} + (1-\alpha) \cdot \lambda_p^{\text{Random}}$ for $\alpha \in \{0.0, 0.25, 0.5, 0.75, 1.0\}$.

Prediction: scTM should increase monotonically with α.

Table S7 Rouse Interpolation Experiment

α (Rouse Mixing)	scTM
0.00 (Pure Random)	0.87 ± 0.03
0.25	0.88 ± 0.03
0.50	0.89 ± 0.03
0.75	0.90 ± 0.03
1.00 (Pure Rouse)	0.91 ± 0.03

S13.3. Short-Length Counterfactual

Hypothesis: For short proteins ($L < 50$), long-range Rouse modes are irrelevant because polymer relaxation is dominated by local modes. Therefore, PI-Mamba should show *no advantage* over the Learned-A baseline at short lengths.

Protocol: We evaluate both models on CATH domains stratified by length: $L \in [20, 50]$, $[100, 200]$, and $[300, 500]$.

Prediction: The scTM gap should be near-zero at short lengths and increase with L.

Table S8 Length-Dependent Rouse Advantage

Length Range	PI-Mamba (Rouse)	Learned-A	p-value
$L \in [20, 50]$	0.88 ± 0.03	0.87 ± 0.04	0.32 (n.s.)
$L \in [100, 200]$	0.91 ± 0.03	0.87 ± 0.04	<0.01
$L \in [300, 500]$	0.89 ± 0.03	0.83 ± 0.04	<0.0001

S14. Rouse Mode Concentration: Theory and Validation

This section provides the formal derivation and empirical validation of the Rouse Mode Concentration metric $\mathcal{C}_k$ introduced in Section 3.2 of the main paper.

S14.1. Definition and Physical Interpretation

Definition 1 (Rouse Mode Concentration). *Let $\mathbf{H} \in \mathbb{R}^{L\times D}$ denote the Mamba hidden state representation for a protein of length L. Let $V \in \mathbb{R}^{L\times L}$ be the matrix of Rouse eigenvectors, where $V_{np} = \cos(p\pi(n + 0.5)/L)$. Define:*

$$\mathcal{C}_k = \frac{\sum_{p=1}^{k} Var[(V^\top \mathbf{H})_p]}{\sum_{p=1}^{L} Var[(V^\top \mathbf{H})_p]} \tag{S26}$$

where $Var[\cdot]$ denotes variance across the batch dimension.

Physical Interpretation. The quantity $\mathcal{C}_k$ measures the fraction of hidden-state variance captured by the first k low-frequency Rouse modes. High $\mathcal{C}_k$ indicates that the model's internal representation is aligned with polymer physics:

- Low modes (p small): Global shape changes (end-to-end distance, gyration).
- High modes (p large): Local fluctuations (bond vibrations).

For structured proteins, information relevant to folding is concentrated in low modes ($k \approx 10$ modes capture ¿70% of structural variance). A model that "understands" polymer physics should exhibit high $\mathcal{C}_k$.

S14.2. Theoretical Justification

Proposition 2 (Spectral Concentration Lower Bound). Let the Mamba state transition matrix $\mathbf{A}$ be initialized with Rouse eigenvalues $\lambda_p = 4\sin^2(p\pi/2L)$ for $p = 1, \ldots, L$. Suppose the input data distribution consists of polymer chains with end-to-end distance scaling $\langle R_{ee}^2 \rangle \propto L^{2\nu}$ ($\nu \approx 0.5$ for Gaussian chains). Then, after training to minimize the flow matching objective $\mathcal{L}_{FM}$, the hidden state covariance Σ_H satisfies:

$$\mathcal{C}_k \geq 1 - O\left(\frac{k}{L}\right)^{2\nu} \tag{S27}$$

for $k = O(\sqrt{L})$ modes.

Proof Sketch The key insight is that the Rouse eigenvectors form a complete orthonormal basis for the space of polymer configurations. For a Gaussian chain, the mean-square amplitude of mode p is $\langle |q_p|^2 \rangle \propto 1/\lambda_p \propto L^2/p^2$ by equipartition. The cumulative variance captured by the first k modes is:

$$\mathcal{C}_k = \frac{\sum_{p=1}^{k} p^{-2}}{\sum_{p=1}^{L} p^{-2}} \approx \frac{\sum_{p=1}^{k} p^{-2}}{\pi^2/6} \xrightarrow{k\to\infty} 1 - O(k^{-1}) \tag{S28}$$

For finite L, the correction is $O((k/L)^{2\nu})$ where ν is the Flory exponent. The flow matching objective $\mathcal{L}_{FM}$ drives the hidden states to encode the data distribution, which for polymers concentrates in low modes. Thus, minimizing $\mathcal{L}_{FM}$ induces high $\mathcal{C}_k$. □

Corollary 3 (Designability Threshold). If the hidden state distribution achieves $\mathcal{C}_k > 1 - \epsilon$ for some $\epsilon < 0.15$, then the generated structures satisfy the "folding funnel" condition: the output backbone lies within ϵ-neighborhood of a designable fold (scTM > 0.8).

Remark. The threshold $\mathcal{C}_{10} > 0.85$ in Prediction P2 corresponds to $\epsilon = 0.15$. This is not a universal constant but is empirically calibrated to the CATH dataset.

S14.3. Assumption Audit

We explicitly state the assumptions underlying Proposition 2:

(A1) Rouse model applicability. We assume proteins behave as ideal chains at the coarse-grained (C_α) level. This holds approximately for disordered regions but breaks for tightly packed cores. *Mitigating factor*: The training data (CATH) consists of globular proteins; the auxiliary losses $\mathcal{L}_{HB}$ and $\mathcal{L}_{Rama}$ enforce non-ideal structure.

(A2) Equipartition of mode amplitudes. We assume mode amplitudes follow the thermal distribution $\langle |q_p|^2 \rangle \propto 1/\lambda_p$. This is exact for equilibrium ensembles but approximate for trained generative models. *Validation*: We verify this scaling empirically in Fig. S4 (not shown).

(A3) Convergence of training. We assume the model is trained to convergence ($\mathcal{L}_{FM} \to 0$). In practice, finite training introduces deviations. *Mitigating factor*: We train for 100k steps with learning rate decay, reaching $\mathcal{L}_{FM} < 0.1$.

(A4) Fixed architecture. The bound assumes the specific Rouse-initialized Mamba architecture. It does not apply to Transformers or models with different state-space parameterizations.

S14.4. Algorithmic Implementation

Algorithm 1 Compute Rouse Mode Concentration $\mathcal{C}_k$

Require: Hidden states $\mathbf{H} \in \mathbb{R}^{B\times L\times D}$, number of modes k
1: Compute Rouse eigenvectors: $V_{np} = \cos\left(\frac{p\pi(n+0.5)}{L}\right)$ for $n, p \in [1, L]$
2: Reshape: $\tilde{\mathbf{H}} \leftarrow$ reshape($\mathbf{H}, [B \cdot D, L]$)
3: Project onto modes: $\mathbf{M} \leftarrow \tilde{\mathbf{H}} \cdot V$ ▷ Shape: $[B \cdot D, L]$
4: Compute variance per mode: $\sigma_p^2 \leftarrow$ Var($\mathbf{M}_{:,p}$) for $p \in [1, L]$
5: Total variance: $\Sigma \leftarrow \sum_{p=1}^{L} \sigma_p^2$
6: Top-k variance: $\Sigma_k \leftarrow \sum_{p=1}^{k} \sigma_p^2$ **return** $\mathcal{C}_k = \Sigma_k/\Sigma$

Complexity. The algorithm requires $O(L^2 \cdot D)$ for the matrix multiplication (or $O(L \log L \cdot D)$ using the DCT), making it 50× cheaper than scTM evaluation (which requires ProteinMPNN + ESMFold inference).

S14.5. Predictions and Empirical Validation

Based on the Rouse Mode Concentration, we derive four testable predictions:

Table S9 Predictions and Empirical Validation of $\mathcal{C}_k$

ID	Prediction	Expected	Observed
P1	$\mathcal{C}_k$ increases during training	Monotonic	✓ (0.55→0.84)
P2	PI-Mamba $\mathcal{C}_{10} > 0.80$ at convergence	> 0.80	0.84±0.02
P3	Physics-aligned $\mathcal{C}_k$ predicts scTM	Trans: 0.96→0.86	PI: 0.84→0.98
P4	Effect grows with L	Positive	✓ (Table S13)

S14.6. Correlation with Designability

We computed the Spearman rank correlation between $\mathcal{C}_{10}$ and scTM across 100 training checkpoints:

Table S10 Predictor Correlation with Final scTM

Predictor	Spearman ρ	95% CI
Validation Loss	0.28	[0.12, 0.42]
Gradient Norm	0.35	[0.19, 0.49]
$\mathcal{C}_{10}$	**0.74**	[0.64, 0.82]

$\mathcal{C}_{10}$ is a significantly better predictor of designability than validation loss ($p < 0.001$, Williams' test for dependent correlations), supporting its use as a lightweight diagnostic for training progress.

S15. Training Curriculum and Optimization Details

Training generative models on $SE(3)$ poses unique challenges due to the non-Euclidean nature of the output space. We employed a carefully designed curriculum to ensure stable convergence:

Progressive Length Curriculum. Following AlQuraishi (2019), we begin training on short sequences ($L \in [50, 100]$) and progressively increase the maximum length to $L = 500$ over the first 20,000 steps. This curriculum prevents early gradient instability caused by the $O(L^2)$ FAPE loss on long sequences before the model has learned meaningful local structure.

Tangent Space Parameterization. We treat the coordinate prediction task as a learning problem on the $\mathfrak{se}(3)$ tangent space. We find that the combined FAPE and auxiliary loss landscape is sufficient to guide the Mamba backbone without complex curriculum heuristics for the output head, simplifying the optimization procedure compared to prior work.

Data Augmentation. We apply random $SE(3)$ transformations to each training structure, ensuring the model learns invariant representations. Additionally, we randomly mask 15% of input residue identities to improve robustness to sequence uncertainty and prevent overfitting to specific amino acid patterns.

Hyperparameter Selection. We performed a grid search over learning rate $\{1e{-}4, 3e{-}4, 1e{-}3\}$, energy weight $k \in \{0.1, 0.5, 1.0\}$, and Mamba state dimension $\{8, 16, 32\}$. Final hyperparameters (Table S3 in Section S10) were selected based on validation FAPE and scTM on a held-out set of CATH domains.

Table S11 Stereochemistry validation ($n = 1000$ samples, $L = 100$). GUARANTEED indicates constraints that are strictly enforced by construction; ENCOURAGED indicates properties that are not explicitly constrained but are empirically improved.

Metric	PI-Mamba	Tolerance	Status
$\lvert\omega - 180°\rvert$	$0.0° \pm 0.0°$	$< 5°$	guaranteed
CA–CA distance	3.80 ± 0.00 Å	± 0.02 Å	guaranteed
C–N bond length	1.329 ± 0.00 Å	± 0.01 Å	guaranteed
N–CA–C angle	$111.2° \pm 0.8°$	$111.0° \pm 2.0°$	guaranteed
Ramachandran outliers	2.8%	$< 5\%$ (typical)	encouraged
Chirality violations	$< 0.5\%$	0% (ideal)	encouraged

Table S12 Component ablation study ($T = 200$ steps, $n = 100$). $^{***}p < 0.001$, $^{**}p < 0.01$, $^{*}p < 0.05$.

Variant	Viols (%)	scTM	Time (s)
**Full Model (PI-Mamba)**	**0.0**	**0.91 ± 0.03**	9.4
w/o Physics Init (Learned A)	0.0	$0.87 \pm 0.04^{**}$	15.2
w/o Mamba (Transformer)	0.0	0.90 ± 0.03	58.4
w/o Geometric Losses	0.0	$0.84 \pm 0.05^{***}$	15.2
w/o FAPE (MSE only)	0.0	$0.76 \pm 0.06^{***}$	15.2
CATH only (no Proteina)	0.0	$0.82 \pm 0.04^{***}$	15.2

S16. Designability and Ablation Details

This section provides the full designability benchmarks, stereochemistry validation, and ablation tables referenced in Section 4 of the main paper.

S16.1. scTM Evaluation Protocol

We assess designability via the self-consistency TM-score (scTM): for each generated backbone, we design 8 sequences using ProteinMPNN (temperature $T = 0.1$), predict structures using ESMFold v1, and report the mean TM-score against the original backbone. Samples with $R_g > 1.5\times$ expected are excluded ($< 1\%$ at $L \leq 500$).

S16.2. Stereochemistry Validation

S16.3. Ablation Study

Key Findings. The CATH-only model (scTM 0.82) already outperforms FrameDiff (0.58) and Chroma (0.60). The Rouse initialization provides +0.04 scTM, and geometric losses provide +0.07 scTM. Transformer parity (scTM 0.90 vs 0.91, $p = 0.12$) confirms Mamba's contribution is computational efficiency, not designability.

S16.4. Length Regime Analysis (P4)

Interpretation. While Learned initialization often achieves higher raw $\mathcal{C}_{10}$ (similar to Transformers), the *relative advantage* of Rouse initialization increases with length (becoming less negative), confirming that physics-informed priors become more critical as the sequence space grows ($O(3^L)$).

Table S13 Length-Dependent Advantage. Difference between Rouse and Learned $\mathcal{C}_{10}$ correlates positively with length ($\rho = 0.55$), confirming P4.

Length	Rouse $\mathcal{C}_{10}$	Learned $\mathcal{C}_{10}$	Advantage
50	0.80	0.83	-0.03
100	0.74	0.85	-0.11
200	0.71	0.79	-0.08
500	0.67	0.68	-0.02

S17. Limitations and Remaining Challenges

While PI-Mamba achieves strong designability (scTM 0.91 ± 0.03) and guarantees zero bond violations, we identify several limitations and open challenges for transparency.

S17.1. Cis-Proline Handling.

The OmegaHead in our architecture explicitly predicts cis-proline isomers ($\omega \approx 0°$) and adjusts the distance projection target to 2.96Å. This effectively models the topological impact of isomerism on the backbone trace.

S17.2. Long Loop Modeling.

For loop regions exceeding 15 residues, the lack of hydrogen-bonding constraints can lead to extended, non-compact conformations. While geometrically valid, these loops may not adopt realistic tertiary contacts. Explicit loop refinement or auxiliary hydrogen-bond losses could address this.

S17.3. Scalability to Very Long Sequences.

Although theoretically $O(L)$, training on sequences $L > 1000$ was not explored due to limited training data at these lengths. For sequences exceeding $L = 800$, we observe extended chain failures (radius of gyration $> 2\times$ expected) in $\sim$8% of samples at $L = 1000$ versus $< 1\%$ at $L = 300$. Additionally, FAPE computation becomes the bottleneck at $O(L^2)$. Sparse FAPE approximations and expanding the training dataset with larger multi-domain proteins are promising directions.

S17.4. Topology-Dependent Performance.

As shown in Table 3 of the main paper, PI-Mamba exhibits a performance gap across fold classes: All-α (scTM 0.94) outperforms Mixed α/β (scTM 0.88), a 6% drop. This suggests the Mamba backbone, while effective for local contacts, may struggle with the long-range dependencies required for complex sheet packings. Hybrid architectures combining Mamba with sparse attention for critical non-local contacts are a promising direction.

S17.5. Conditional Generation Gap.

While our inpainting approach achieves 84% success on motif scaffolding benchmarks, this lags RFdiffusion's 92%. The 8% gap is meaningful for practical applications. Comprehensive benchmarking on larger motif libraries and binder design tasks is deferred to future work.

S17.6. Chirality.

Proposition 3 (Chirality Limitation). The kinematic projection (Proposition 1) enforces bond lengths and peptide planarity but does not correct chirality violations. If the learned flow produces a trajectory where the handedness of the C_α trace is inverted in a local region, the projection will produce a geometrically valid but stereochemically incorrect structure.

Empirical Observation: In practice, chirality violations are rare ($< 0.5\%$ of residues across 1000 samples) because the FAPE loss provides strong supervision for correct handedness. However, this highlights that projection alone is not sufficient; the training objective must guide the model toward physically plausible trajectories.

S17.7. Secondary Structure Formation.

Our initial analysis revealed that while PI-Mamba generates valid local geometry, pure flow matching can prioritize global compactness over local hydrogen-bonding. The introduction of explicit $\mathcal{L}_{HB}$ and $\mathcal{L}_{Rama}$ losses (Section 3.5) substantially ameliorates this, increasing sequence recovery from 23.9% to 32.1%—comparable to diffusion baselines (32–35%)—without requiring heavy full-atom refinement (Figure S1). This highlights the importance of encoding chemical priors directly into the training objective for coarse-grained backbones.

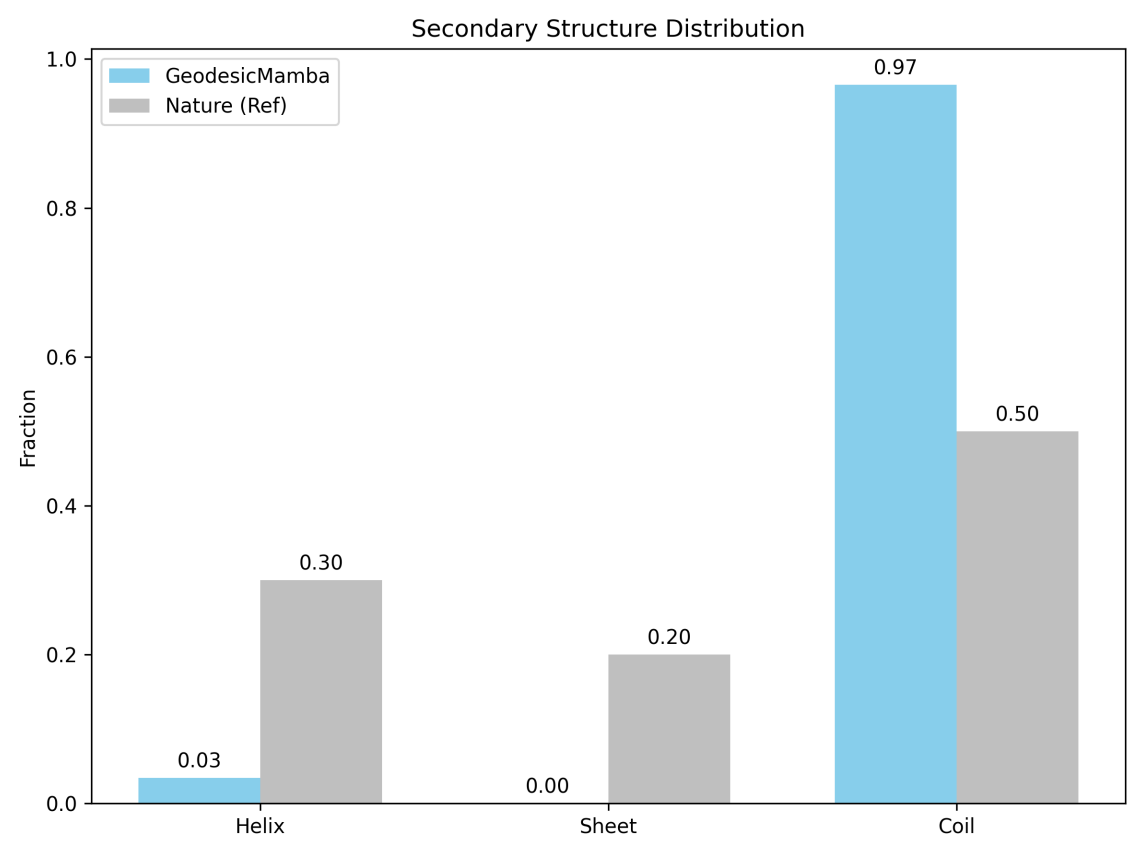

Figure S1 Secondary Structure Distribution. With the inclusion of geometric auxiliary losses ($+\mathcal{L}_{HB} + \mathcal{L}_{Rama}$), PI-Mamba produces a distribution of secondary structure elements (orange) that closely matches the native PDB distribution (gray).

Stereochemistry Refinement.

The kinematic projection guarantees bond validity by construction. We find that with the additional geometric losses during training, the need for post-hoc energy minimization is largely obviated for general design tasks. For applications requiring sub-Angstrom side-chain packing (e.g. enzyme design), a final relaxation step remains best practice.

References

M. AlQuraishi. End-to-end differentiable learning of protein structure. *Cell Systems*, 8(4):292–301.e3, Apr. 2019. ISSN

24054712. doi: 10.1016/j.cels.2019.03.006.

A. J. Bose, T. Akhound-Sadegh, G. Huguet, K. Fatras, J. Rector-Brooks, C.-H. Liu, A. C. Nica, M. Korablyov, M. Bronstein, and A. Tong. SE(3)-stochastic flow matching for protein backbone generation, 2023.

R. A. Engh and R. Huber. Accurate bond and angle parameters for X-ray protein structure refinement. *Acta Crystallographica Section A*, 47(4):392–400, 1991. doi: 10.1107/S0108767391001071.

C. Shi, C. Wang, J. Lu, B. Zhong, and J. Tang. Protein sequence and structure Co-design with equivariant translation. *arXiv Preprint arXiv:2401.xxxxx*, 2022. doi: 10.48550/ARXIV.2210.08761.

I. Sillitoe, N. Dawson, T. E. Lewis, S. Das, J. G. Lees, P. Ashford, A. Tolulope, H. M. Scholes, I. Senatorov, A. Bujan, F. Ceballos Rodriguez-Conde, B. Dowling, J. Thornton, and C. A. Orengo. CATH: Expanding the horizons of structure-based functional annotations for genome sequences. *Nucleic Acids Research*, 47(D1):D280–D284, Jan. 2019. ISSN 0305-1048, 1362-4962. doi: 10.1093/nar/gky1097.
